# Path analyses of risk factors for linear growth faltering in four prospective cohorts of young children in Ghana, Malawi and Burkina Faso

**DOI:** 10.1136/bmjgh-2018-001155

**Published:** 2019-01-13

**Authors:** Elizabeth L Prado, Elizabeth Yakes Jimenez, Stephen Vosti, Robert Stewart, Christine P Stewart, Jérôme Somé, Anna Pulakka, Jean Bosco Ouédraogo, Harriet Okronipa, Eugenia Ocansey, Brietta Oaks, Kenneth Maleta, Anna Lartey, Emma Kortekangas, Sonja Y Hess, Kenneth Brown, Jaden Bendabenda, Ulla Ashorn, Per Ashorn, Mary Arimond, Seth Adu-Afarwuah, Souheila Abbeddou, Kathryn Dewey

**Affiliations:** 1 Department of Nutrition, University of California Davis, Davis, California, USA; 2 Departments of Pediatrics and Internal Medicine, University of New Mexico Health Sciences Center, Albuquerque, New Mexico, USA; 3 Department of Agricultural and Resource Economics, University of California Davis, 2135 Social Sciences and Humanities, Davis, California, USA; 4 Department of Psychiatry, University of Malawi College of Medicine, Blantyre, Southern Region, Malawi; 5 Institut de Recherche en Sciences de la Santé, Avenue de la Liberté, Burkina Faso; 6 Department of Public Health, University of Turku and Turku University Hospital, Finland, Turku; 7 Department of Nutrition and Food Sciences, University of Rhode Island, Kingston, Rhode Island, USA; 8 School of Public Health and Family Medicine, University of Malawi College of Medicine, Blantyre, Malawi; 9 Department of Nutrition and Food Science, University of Ghana, Legon, Ghana; 10 Center for Child Health Research, University of Tampere School of Medicine and Tampere University Hospital, University of Tampere, Tampere, Finland; 11 Bill & Melinda Gates Foundation, Seattle, Washington, USA; 12 Department of Paediatrics, Tampere University Hospital, Tampere, Finland; 13 Intake, Center for Dietary Assessment, Seattle, Washington, USA

**Keywords:** Linear growth, stunting, path analysis, prospective cohort, Africa, pregnancy, childhood

## Abstract

Stunting prevalence is an indicator of a country’s progress towards United Nations’ Sustainable Development Goal 2, which is to end hunger and achieve improved nutrition. Accelerating progress towards reducing stunting requires a deeper understanding of the factors that contribute to linear growth faltering. We conducted path analyses of factors associated with 18-month length-for-age z-score (LAZ) in four prospective cohorts of children who participated in trials conducted as part of the International Lipid-Based Nutrient Supplements Project in Ghana (n=1039), Malawi (n=684 and 1504) and Burkina Faso (n=2619). In two cohorts, women were enrolled during pregnancy. In two other cohorts, infants were enrolled at 6 or 9 months. We examined the association of 42 indicators of environmental, maternal, caregiving and child factors with 18-month LAZ. Using structural equation modelling, we examined direct and indirect associations through hypothesised mediators in each cohort. Out of 42 indicators, 2 were associated with 18-month LAZ in three or four cohorts: maternal height and body mass index (BMI). Six factors were associated with 18-month LAZ in two cohorts: length for gestational age z-score (LGAZ) at birth, pregnancy duration, improved household water, child dietary diversity, diarrhoea incidence and 6-month or 9-month haemoglobin concentration. Direct associations were more prevalent than indirect associations, but 30%–62% of the associations of maternal height and BMI with 18-month LAZ were mediated by LGAZ at birth. Factors that were not associated with LAZ were maternal iron status, illness and inflammation during pregnancy, maternal stress and depression, exclusive breast feeding during 6 months post partum, feeding frequency and child fever, malaria and acute respiratory infections. These findings may help in identifying interventions to accelerate progress towards reducing stunting; however, much of the variance in linear growth status remained unaccounted for by these 42 individual-level factors, suggesting that community-level changes may be needed to achieve substantial progress.

**Key question**

**What is already known?**

- Previous cross-sectional studies have identified key risk factors for stunted growth, including being small for gestational age at birth, poor sanitation, childhood diarrhoea, and low dietary diversity and infrequent infant feeding.

**What are the new findings?**

- We found consistent associations of 18-month length-for-age z-scores (LAZ) with maternal height and maternal body mass index.
- The factors with the strongest associations with 18-month LAZ were length for gestational age z-score (LGAZ) at birth, maternal height and gestational age at birth.
- Other factors that showed significant associations with 18-month LAZ (although with smaller coefficients), were improved household water source, child dietary diversity, childhood diarrhoea incidence and 6-month or 9-month haemoglobin concentration.

**What do the new findings imply?**

- Interventions targeting these factors associated with LAZ may accelerate progress towards reducing stunting; however, much of the variance in linear growth status remained unaccounted for by individual-level factors, suggesting that community-level changes may be needed to achieve substantial progress and further research is needed to understand the causes of stunting.

## Introduction

Linear growth faltering during early life is associated with later health conditions, such as cardiometabolic disease,^1^ and poor cognitive and school performance.^2^ Stunting occurs when children falter in linear growth early in life and is defined as height-for-age >2 SD below the median of the WHO Child Growth Standards.^3^ Stunting prevalence is an important indicator to evaluate a country’s progress towards United Nations’ Sustainable Development Goal 2, which is to end hunger and achieve improved nutrition by 2030. From 2000 to 2017, the estimated number of stunted children under 5 years of age globally decreased from 198 million to 151 million.^4^ Accelerating progress towards reducing the prevalence of stunting requires a more advanced understanding of the factors that contribute to early linear growth faltering.

Several previous reviews have summarised these risk factors,^5–7^ identifying environmental factors such as household food insecurity and poor quality water and sanitation; maternal factors such as short stature and poor nutrition and health during pregnancy; caregiving factors, such as infrequent feeding and low dietary diversity; and child factors such as being born preterm, small for gestational age and childhood diarrhoea incidence. Frameworks that describe the determinants of childhood malnutrition^5 6^ recognise that distal factors (eg, community, household level) operate through more proximal factors (eg, maternal, child level) in their influence on growth faltering. However, most studies that have attempted to quantify the relative contribution of risk factors for linear growth faltering have used cross-sectional analyses, such as the estimation of regression coefficients^8–10^ or population-attributable fraction.^11 12^ These methods describe direct associations, but do not account for the indirect associations of distal factors through proximal factors.

In the current study, we performed a set of path analyses of factors associated with linear growth status at age 18 months in four longitudinal cohorts of children (n*=*5846) who participated in the International Lipid-Based Nutrient Supplements (iLiNS) Project in Ghana, Malawi and Burkina Faso. The first objective was to identify the factors associated with 18-month linear growth status, reflecting cumulative linear growth during gestation and the first 18 months after birth, in each iLiNS cohort. The second objective was to identify the pathways through which these factors operate. The prospective design enabled examination of factors associated with linear growth throughout most of the first 1000 days after conception. Harmonisation of many of the variables across cohorts allowed examination of the same factors and pathways in different contexts.

Finally, our third objective was to determine whether a pooled analysis examining predictors of 18-month length-for-age z-score (LAZ) across cohorts accounted for more variance in LAZ than the within-cohort models. Evidence suggests that linear growth faltering is a whole-population phenomenon. In populations with a high prevalence of stunting, it is not the case that a subgroup of that population is faltering while the rest are growing normally, but instead there is usually a downward shift in the entire height distribution of the population.^13^ If between-population differences are more meaningful than within-population differences between individuals, then we would expect the pooled analysis to account for more variance than the analyses of each separate cohort.

## Methods

**Figure 1:**
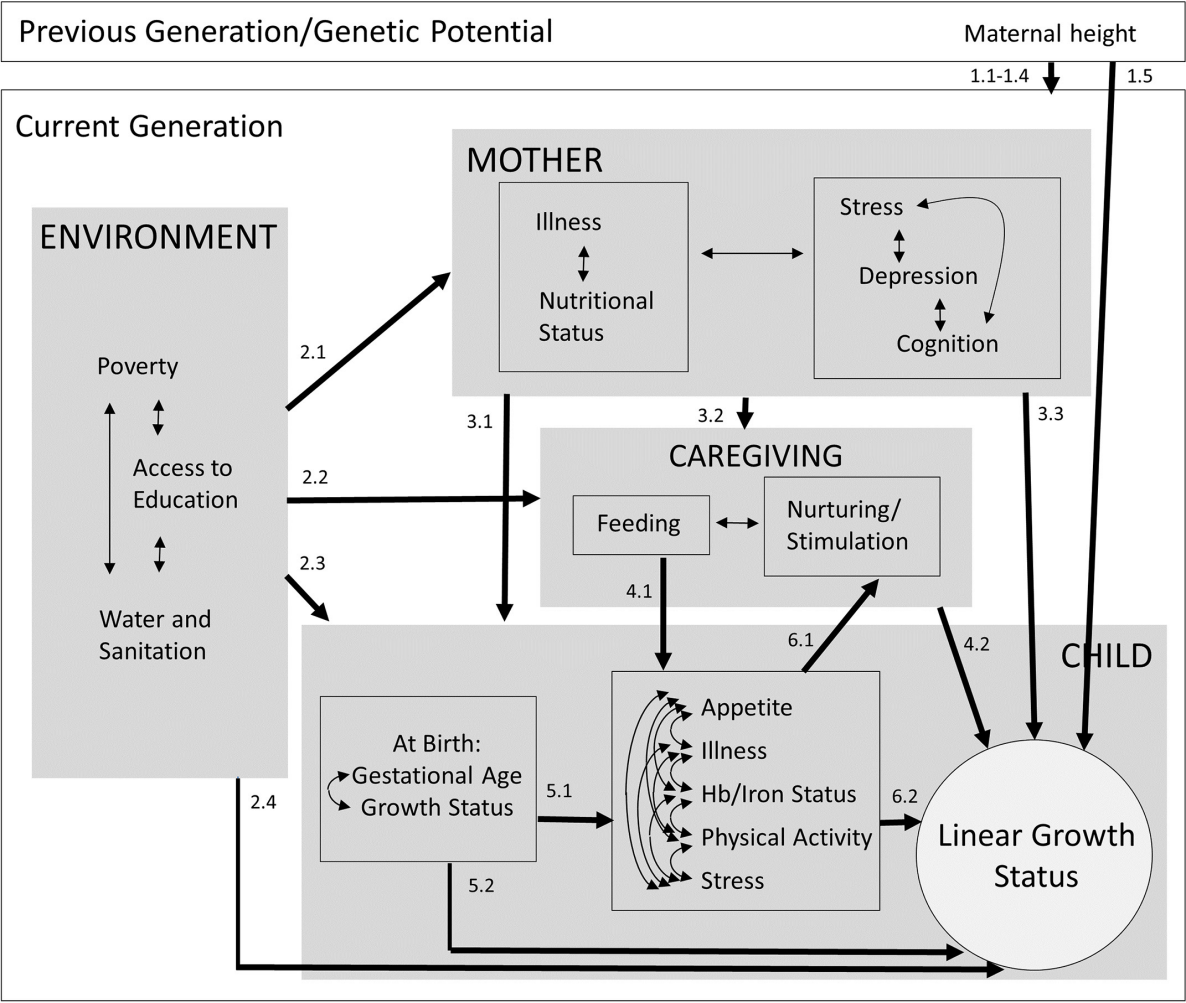
Conceptual model.

Based on several previous frameworks,^5 6 14^ we developed a conceptual path model of potential influences on 18-month linear growth status (figure 1). We tested the following pathways, which correspond to the labels of the arrows in figure 1.

### Pathway (1)

At an individual level, maternal height may be related to the child’s genetic potential for adult height that can be attained. In populations with a high prevalence of stunting, maternal height is also partly a reflection of growth restriction experienced by the mother during early life. Therefore, inclusion of maternal height in the model served two purposes: first, to adjust for a proxy of genetic potential, and second, to test the pathway that intergenerational effects of maternal growth during early life, reflected by maternal adult height, on child linear growth may be mediated by (1.1) socioeconomic conditions of the current generation, (1.2) maternal adult factors (nutritional status, illness, stress, depression, cognition), (1.3) caregiving practices, (1.4) child factors or (1.5) may directly affect linear growth (arrows for each individual pathway not drawn in the figure).

### Pathway (2)

Socioeconomic disparities and other environmental effects on child linear growth may be mediated by (2.1) maternal factors, (2.2) caregiving practices, (2.3) child factors or (2.4) may directly affect linear growth.

### Pathway (3)

Effects of maternal factors on child growth may be mediated by (3.1) child factors, (3.2) caregiving practices or (3.3) may directly affect child growth.

### Pathway (4)

Effects of infant feeding practices on child growth may be mediated by child factors (4.1) or caregiving practices may directly affect child growth (4.2).

### Pathway (5)

Effects of preterm birth or intrauterine growth restriction on later child linear growth status may be mediated by (5.1) postnatal child factors (appetite, illness, haemoglobin (Hb)/iron status, physical activity, stress) or (5.2) may be direct effects.

### Pathway (6)

Effects of child factors (appetite, illness, Hb/iron status, physical activity, stress) on child growth (6.1) may be mediated by caregiving behaviour in response to these factors, or (6.2) may be direct effects.

#### iLiNS project trial designs

In the iLiNS-DYAD-G trial in Ghana (n=1320) and the iLiNS-DYAD-M trial in Malawi (n=869), pregnant women were enrolled at ≤20 weeks of gestation. In the iLiNS-DOSE trial in Malawi (n=1932) and iLiNS-ZINC trial in Burkina Faso (n=3220), infants were enrolled at age 6 and 9 months, respectively. All participants were assigned to receive various doses and formulations of lipid-based nutrient supplements (LNS), or to control groups until age 18 months, when length was measured.^15–18^ The effects of the interventions on 18-month child length-for LAZ differed across trials, with positive effects in Burkina Faso^17^ and Ghana,^15^ but not in Malawi.^16 18^ For further information, see supplemental methods.

#### Participants

In the path analyses reported here, we included all children for whom LAZ at age 18 months was available, comprising 1039 children in iLiNS-DYAD-G, 684 in iLiNS-DYAD-M, 1504 in iLiNS-DOSE and 2619 children in iLiNS-ZINC.

#### Procedure

**Figure 2:**
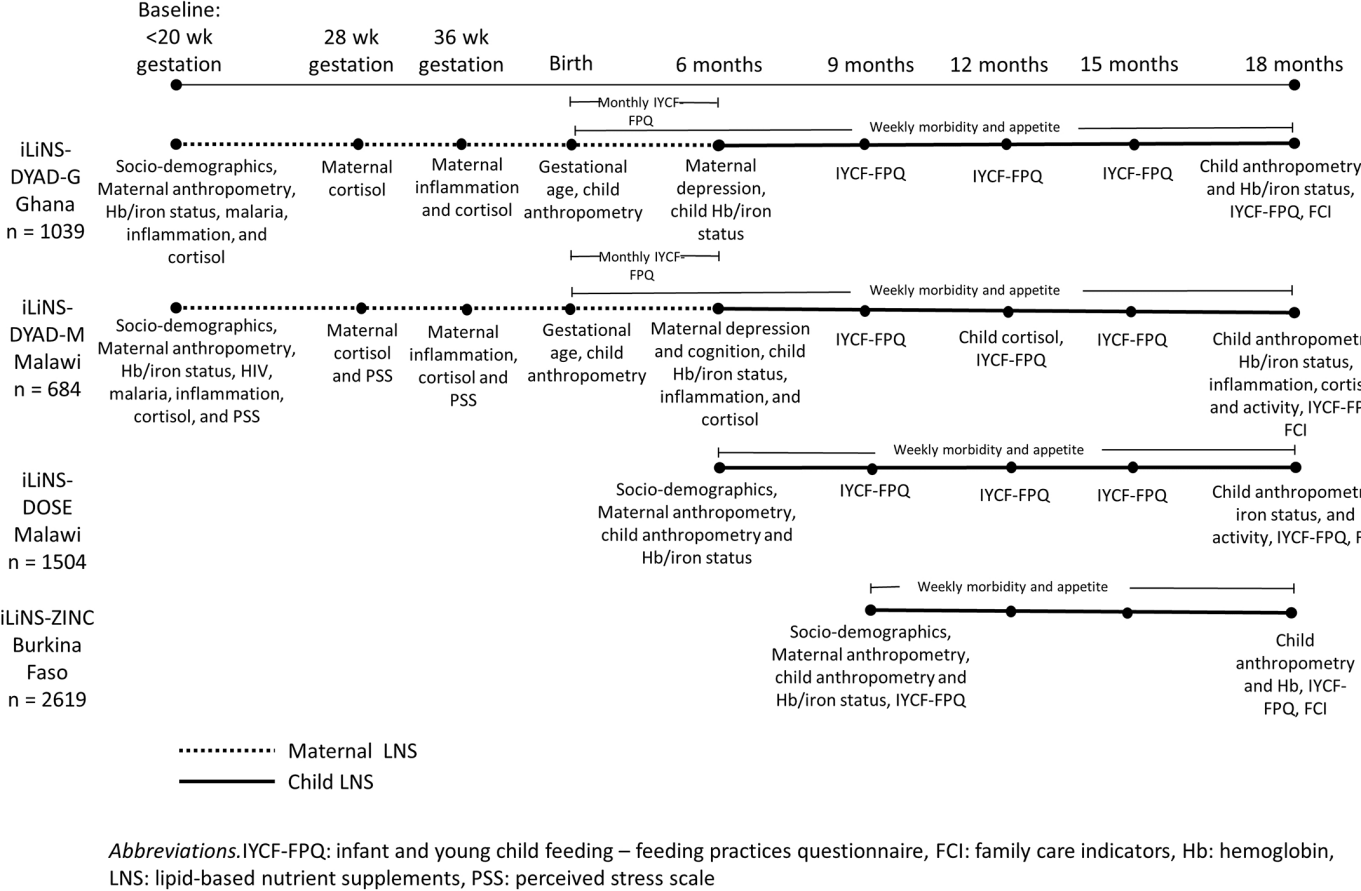
Data collection timeline in each cohort.

Detailed reports of the data collection procedures in each trial have been published elsewhere^15–18^; therefore, we summarise the procedures for collection of variables that were used in the analyses presented here. Online supplementary table 1 presents further details of the data collection procedures and variable definitions. Figure 2 shows the data collection schedule for each variable in each cohort.

10.1136/bmjgh-2018-001155.supp1 Supplementary data

Data on socio-demographic characteristics and maternal anthropometric status were gathered at enrolment. In the two DYAD trials, maternal pre-pregnancy body mass index (BMI) was estimated based on BMI and gestational age at enrolment. Capillary or venous blood samples were collected from mothers and/or children at multiple time points for the assessment of (1) malaria using a rapid diagnostic test, (2) Hb concentration (g/L), (3) biomarkers of iron status, including zinc protoporphyrin (ZPP) concentration (μmol/mol heme), and soluble transferrin receptor (sTfR, mg/L), and (4) biomarkers of inflammation, including alpha-1-acid glycoprotein (AGP; g/L) concentration. In ZINC, known HIV infection was an exclusion criterion, but HIV was not tested; therefore, HIV status of women who were enrolled was unknown. In DOSE, HIV status was also unknown. In DYAD-G, HIV infection was known from antenatal cards and HIV-positive women were excluded. In DYAD-M, women were tested for HIV at enrolment but were not excluded.

Maternal and/or child saliva samples in DYAD-M and DYAD-G were collected at several time points for the measurement of cortisol concentration (nmol/L). Maternal self-reported stress was measured in DYAD-M at multiple time points using the Perceived Stress Scale.^19 20^ Mothers were interviewed regarding depressive symptoms at 6 months post partum in DYAD-M using a locally validated adaptation of the Self-Reporting Questionnaire and in DYAD-G with the Edinburgh Post-natal Depression Scale.^21^ In DYAD-M, at 6 months post partum, maternal cognition was assessed using digit span forward and backward, verbal fluency, mental rotation and functional health literacy tests.^22^

Children were visited weekly for morbidity surveillance. At these visits, caregivers were asked whether the child experienced any illness symptoms, including fever, diarrhoea, vomiting, cough, nasal discharge, respiratory distress or poor appetite during the past seven days and/or data collectors measured the child’s auricular temperature. Longitudinal prevalence and/or incidence of diarrhoea, fever, malaria, acute respiratory infection and/or poor appetite were calculated (online supplementary table 1). In DOSE and DYAD-M, physical activity at age 18 months was measured over 1 week with the hip-worn ActiGraph GT3X+accelerometer (Pensacola, Florida, USA).^23^

Infant feeding practices were assessed at multiple time points through qualitative 24 hours and/or 7-day dietary recall questionnaires.^24^ In all four trials, developmental stimulation was measured at age 18 months using the Family Care Indicators interview.^25^ The mother was interviewed with regard to the variety of play materials and activities that adults used to engage with the child in the past three days.

In DYAD-G and DYAD-M, gestational age at enrolment was mainly determined by ultrasound and this was used to calculate gestational age at birth. In DYAD-G, infant weight and length were measured within 48 hours of birth or between 3 and 14 days after birth for 87 (9.4%) when the former was not possible. In DYAD-M, infant weight and length were measured within 6 weeks of birth. We estimated length and weight at birth based on LAZ and WAZ measured within 6 weeks of birth, assuming that LAZ and WAZ did not change from birth to the time of measurement. Length and weight for gestational age at birth z-scores were then calculated based on the INTERGROWTH-21st standards.^26^ In all four cohorts, length was measured at age 18 months. All length measurements were conducted to the nearest 1 mm by teams of two trained and standardised anthropometrists using length boards.

#### Analysis

We examined the distribution of each independent variable separately by cohort. We log-transformed skewed variables and winsorised outliers to the 1st and 99th percentile. If transformation did not result in a normal distribution, we created a binary variable. All continuous variables were standardised to SD units by subtracting the mean and dividing by SD.

**Figure 3:**
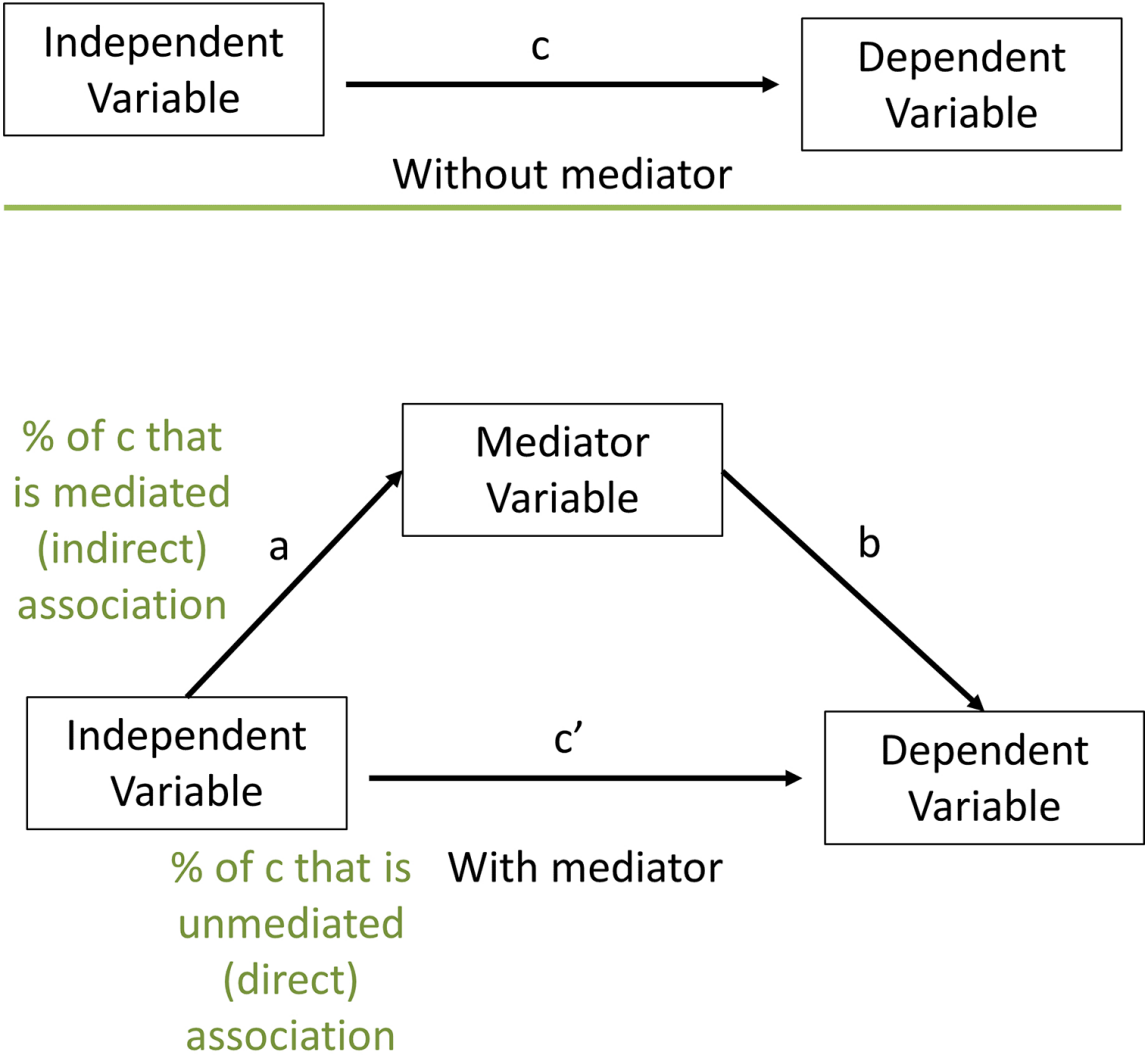
Mediation analysis.

We performed exploratory mediation analyses according to the following steps. We refer to figure 3 to describe each step in the mediation analysis.

##### Step 1: variable selection

The first condition for inclusion in our mediation analysis was that X is associated with Y, represented by *c* in figure 3, so we examined independent associations between each predictor and 18-month LAZ and dropped any that were not associated at p<0.05. The second condition was that X is associated with M, represented by *a*. The third condition was that M is associated with Y, represented by *b*. In this first step, we tested the significance of *b* and *c*.

Second, we reduced the number of variables measuring the same construct by eliminating variables that were collinear. If two variables were highly collinear (*r*>0.6), we dropped the one that was less strongly associated with 18-month LAZ. Third, we examined four multivariate models with each category of factors together predicting 18-month LAZ and dropped any that were not associated at p<0.05. If, at this step, no variable was significantly associated when controlling for the others, we retained the one that was most strongly associated with 18-month LAZ. The four multivariate models were (1) all environmental factors together, (2) all maternal factors together, excluding maternal height, (3) all caregiving factors together and (4) all child factors together, excluding gestational age at birth and LGAZ at birth.

##### Step 2: path selection

For each pathway in figure 1, we examined the association between each pair of variables on the pathway to determine which variables were potential mediators. In this second step, we tested the significance of *a* in figure 3 and dropped any pathways for which p>0.05. In figure 1, unidirectional arrows represent pathways tested. Bidirectional arrows represent associations that were checked for collinearity, but were not otherwise modelled in the path analysis. All analyses up to this point were conducted using SAS V.9.4 (SAS Institute). Next, for each independent variable with potential mediators, we tested the mediation model using Stata V.14.1 (StataCorp) binary mediation program. We ran the multiple mediation model including all potential mediators together, rather than testing each mediator one by one in separate models. In the final path model, we included all pathways for which the indirect association of X with Y through M was significant. If the interaction between X and M was significant at p<0.05, we stratified the sample at the median of the independent variable and tested the indirect association of X through M at both high and low levels of X. If the indirect association was significant at both high and low levels of X, then we retained the pathway in the model; otherwise, we removed that pathway.

Finally, we ran the final path model using the *sem* command in Stata with the *mlmv* option to estimate the model on the full data set using maximum likelihood estimation for missing values. All models controlled for three covariates: randomly assigned trial group (LNS vs no LNS), child sex and child age at 18-month LAZ assessment. In the final models, we corrected p values for multiple comparisons using the Benjamini-Hochberg correction,^27^ which is recommended for controlling the false discovery rate in structural equation models.^28^ We applied the correction separately for each model (ie, for each cohort). If any pathway was not significant at corrected p<0.05, then we did not draw that pathway in the path diagram.

For objective 3, we examined the 16 variables that were available in all four cohorts: household asset index, household food insecurity access index, maternal and paternal education, household water and toilet, maternal age, height, and BMI, child diarrhoea and fever prevalence, child 6-month or 9-month Hb and ZPP, mean dietary diversity across time points, variety of play materials and activities with caregivers at age 18 months. We report the R^2^ in the models with these 16 predictors in each cohort separately and in the pooled model to determine whether the pooled analysis accounted for more variance in 18-month LAZ than the within-cohort models.

## Results

Summary statistics for all independent variables are presented in online supplementary table 2. The variable selection results are shown in online supplementary table 3-6. The pathway selection results are shown in online supplementary table 7-10. At age 18 months, the mean (SD) LAZ in each cohort was −0.8 (1.0) in DYAD-G, −1.7 (1.1) in DYAD-M, −1.9 (1.1) in DOSE and −1.5 (1.1) in ZINC. The percentage of children who were stunted (LAZ < −2) at age 18 months in each cohort was 12% in DYAD-G, 31% in DYAD-M, 43% in DOSE and 32% in ZINC.

Among environmental variables, 18-month LAZ was positively associated with higher household assets (DYAD-G), household being located closer to the market (DOSE), higher maternal education (DOSE, ZINC), higher paternal education (DYAD-M) and an improved water source (DOSE, ZINC). Of the maternal variables, we observed higher LAZ among children of mothers who were taller in all four cohorts and among children of mothers who had higher BMI in three cohorts (DYAD-M, DOSE, ZINC). In DYAD-G, higher LAZ was associated with higher maternal Hb at ≤20 weeks gestation. Among the caregiving variables, we observed higher LAZ among children who had greater dietary diversity (at 15 months in DOSE and at 9 months in ZINC), higher 18-month variety of play materials (DYAD-M, ZINC) or higher 18-month activities with caregivers (DYAD-M).

**Figure 4:**
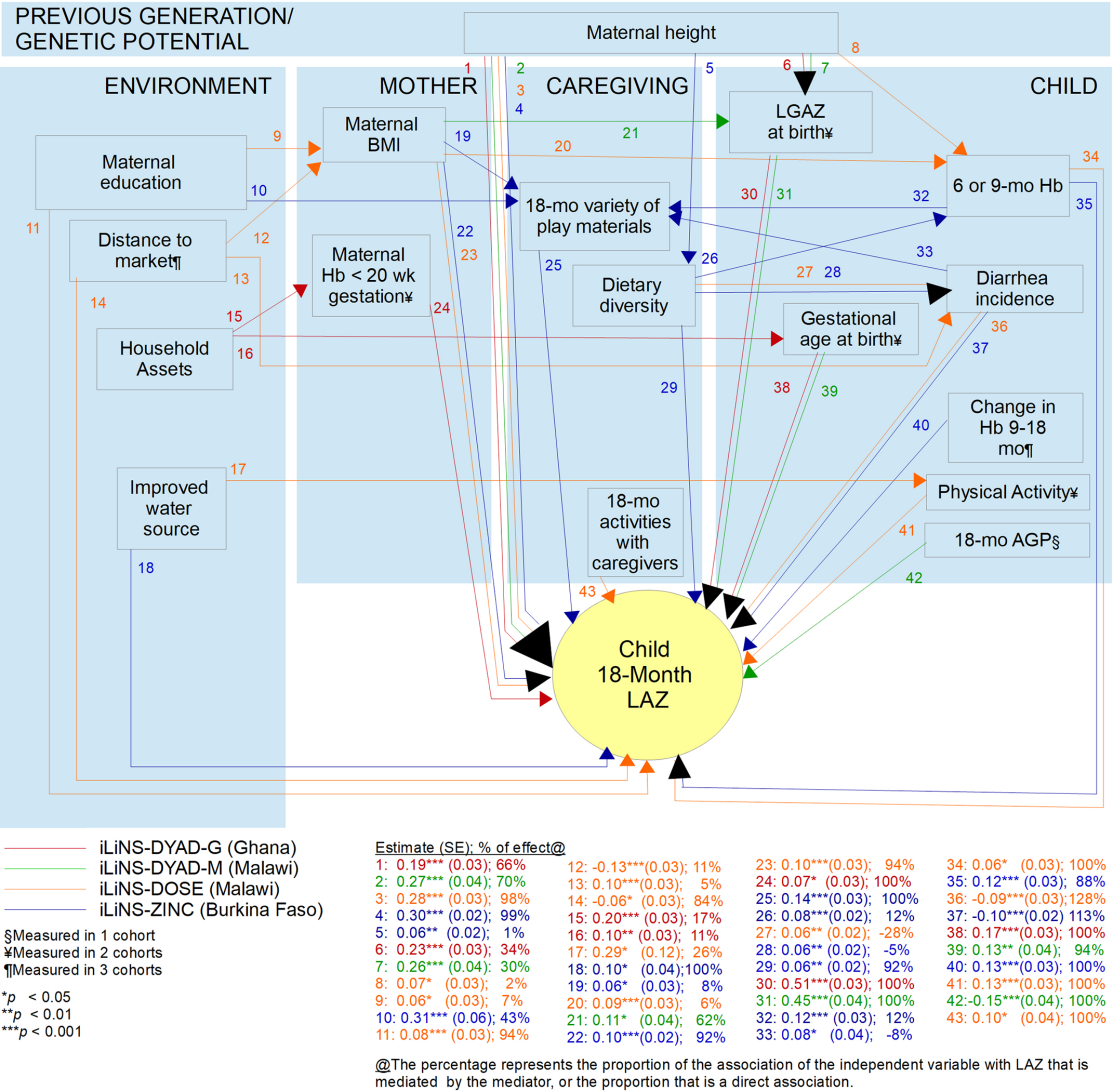
Path diagram of factors associated with18-month LAZ in four iLiNS cohorts. ^1^Measured in one cohort. ^2^Measured in two cohorts. ^3^Measured in three cohorts. AGP, alpha-1-acid glycoprotein; BMI, body mass index; Hb, haemoglobin; iLiNS International Lipid-Based Nutrient Supplements; LAZ, length-for-age z-score.

Of the child variables, higher LAZ was associated with higher Hb at 6 (DYAD-M, DOSE) or 9 months (ZINC), greater positive change in Hb from 9 to 18 months (ZINC), lower 18-month AGP (DYAD-M), lower diarrhoea incidence from 6 (DOSE) or 9 (ZINC) to 18 months, lower prevalence of poor appetite from 6 to 18 months (DYAD-G) and higher activity levels as measured by accelerometer mean vector magnitude at 18 months (DOSE). In the two cohorts enrolled before birth, both gestational age at birth and LGAZ at birth were strongly associated with 18-month LAZ. All coefficients in the final models are presented in figure 4 and online supplementary table 11.

Regarding pathway (1), examination of genetic effects or intergenerational effects of maternal growth during early life, reflected by maternal adult height, on child 18-month LAZ consistently showed that this was largely a direct association (66%–99% of the association between maternal height and LAZ across the four cohorts) rather than via mediation by other factors. In the two cohorts in which length at birth was measured, LGAZ at birth mediated a substantial proportion of this association (34% in DYAD-G, 30% in DYAD-M). A very small proportion of this association was mediated by child Hb at 6 months (2% in DOSE) or dietary diversity in infant feeding (1% in ZINC).

For pathway (2), the extent to which socioeconomic disparities were mediated by other factors varied across cohorts and independent variables, from 0% to 43%. In ZINC, the association of maternal education with 18-month LAZ was mediated by the child’s variety of play materials (43%) and the association of improved water source with LAZ was not mediated by any other variables. In DYAD-G, the association between household assets and child LAZ was partly mediated by maternal lower Hb concentration in early pregnancy (17%) and gestational age at birth (11%). In DOSE, three environmental variables were associated with LAZ: distance to the nearest market, maternal education and an improved water source. The direct proportion of these associations ranged from 74% to 93%. The association of distance to the nearest market with LAZ was partly mediated by maternal BMI (11%) and child diarrhoea incidence (5%). The association of maternal education with 18-month LAZ was partly mediated by maternal BMI (7%). The association of improved water source with child LAZ was partly mediated by child physical activity (26%). In DYAD-M, while the association of paternal education with LAZ was retained in the variable selection process, it was not significant in the final model and was not mediated by any variables.

Regarding pathway (3), the extent to which associations of maternal health and nutritional status with child LAZ were mediated versus direct effects also varied by cohort and independent variable (0%–56%). In DYAD-M, the association between maternal BMI and 18-month LAZ was largely mediated by LGAZ at birth (62%). In the two cohorts in which LGAZ at birth was not available, the association of maternal BMI with LAZ was largely a direct association (92%–94%), with a small proportion mediated by the child’s variety of play materials (8% in ZINC) or child Hb concentration at 6 months (6% in DOSE). In DYAD-G, the association of maternal Hb in early pregnancy with 18-month LAZ was not mediated by any other variables.

Associations of dietary diversity in infant feeding at 9 months in ZINC and 15 months in DOSE with 18-month LAZ (pathway 4.1) were partly mediated by child diarrhoea incidence. Caregivers who reported higher dietary diversity in infant feeding also reported higher diarrhoea incidence in the child. The association of dietary diversity with 18-month LAZ was also positive and the association of diarrhoea incidence with 18-month LAZ was negative; therefore, the proportion of the association between dietary diversity and child LAZ that was mediated by diarrhoea incidence was negative (−5% in ZINC and −28% in DOSE).

Results from both trials that measured gestational age and length at birth showed that these were direct associations, unmediated by any other child factors (pathway 5.2). The following results from the ZINC cohort supported pathway (6.1): children with lower Hb at age 9 months were provided with lower variety of play materials at 18 months, mediating 12% of the association with 18-month LAZ. Similar to dietary diversity, variety of play materials was positively associated with diarrhoea incidence, therefore this mediated −8% of the negative association between diarrhoea incidence and child LAZ.

**Figure 5:**
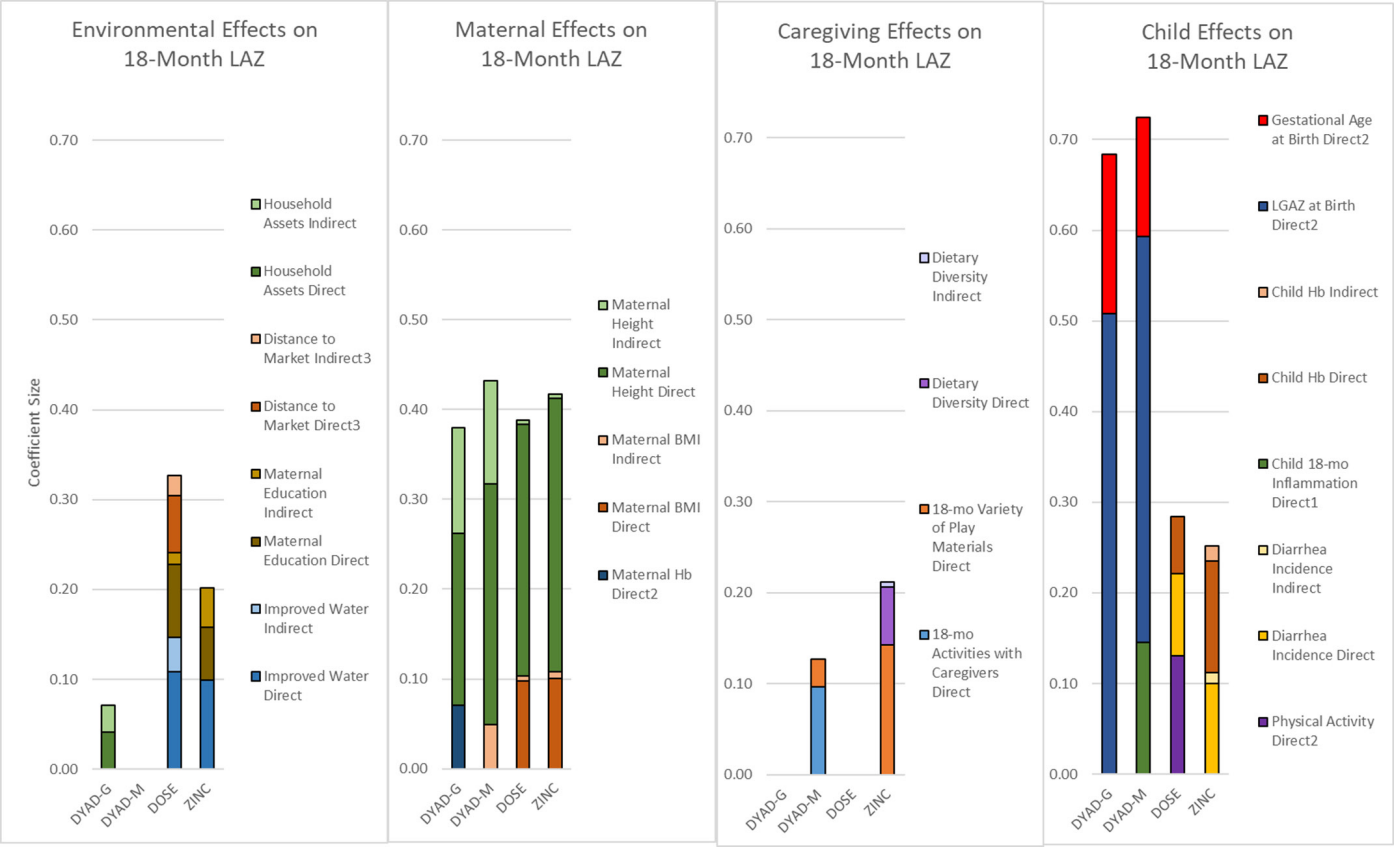
Coefficients of direct and indirect effects on 18-month LAZ in the final structural equation models. BMI, body mass index; Hb, haemoglobin; LAZ, length-for-age z-score.

The final models accounted for 34% of the variance in 18-month LAZ in DYAD-G, 37% in DYAD-M, 17% in DOSE and 20% in ZINC. Overall, maternal and child factors showed stronger associations with 18-month LAZ compared with environmental and caregiving factors, even when considering both indirect and direct effects (figure 5).

For objective 3, the 16 variables common across all four cohorts accounted for the following percentage of variance in 18-month LAZ: 16% in DYAD-G, 21% in DYAD-M, 15% in DOSE, 19% in ZINC, and 25% in the pooled analysis. Of these 16 variables, maternal height accounted for the largest proportion of variance in 18-month LAZ: 11% in DYAD-G, 14% in DYAD-M, 10% in DOSE, 11% in ZINC and 9% in the pooled analysis. Therefore, excluding maternal height, environmental, maternal, caregiving and child factors accounted for only 5%–8% of variance in LAZ in the individual cohorts and 16% in the pooled analysis.

## Discussion

In four prospective cohorts of children, we conducted path analyses of environmental, maternal, caregiving and child factors hypothesised to be associated with 18-month linear growth status. Out of 42 indicators examined, only 2 indicators emerged as consistent predictors of 18-month LAZ in 3–4 cohorts: maternal height and maternal BMI. Two additional factors, which were only measured in the two pregnancy cohorts, were significantly associated with 18-month LAZ in both cohorts in which they were measured: LGAZ at birth and gestational age at birth. In the two cohorts enrolled after birth, four factors were significantly associated with 18-month LAZ in both cohorts: improved household water source, dietary diversity in infant feeding, childhood diarrhoea incidence and 6-month or 9-month Hb. Overall, maternal and child factors showed stronger and more consistent associations with child LAZ than environmental and caregiving factors, even when considering both indirect and direct effects.

While specific pathways differed across cohorts, in general direct associations were stronger and more prevalent than indirect associations. The strongest evidence for mediation was the finding that 62% of the association of maternal BMI with 18-month LAZ was mediated by LGAZ at birth in DYAD-M. This underscores the importance of maternal nutritional status for fetal growth, with lasting consequences for growth status later in childhood. LGAZ at birth also mediated a substantial proportion of the association of maternal height with 18-month LAZ (30%–34%) in both cohorts in which it was measured (DYAD-M and DYAD-G). This pathway is likely to reflect genetic influences, at least in part, and therefore may be only partly modifiable. In the two cohorts in which LAZ at birth was not measured (DOSE and ZINC), the association of maternal height with 18-month LAZ was a direct association.

Of the eight key risk factors closely associated with linear growth faltering that we found, three were consistently identified in previous studies that examined the relative contribution of risk factors to stunting prevalence globally: fetal growth restriction, diarrhoea and low dietary diversity. Danaei *et al* used population surveys to estimate cases of stunting attributable to 18 risk factors in 137 developing countries, including indicators of maternal nutrition and infection, teenage motherhood and short birth intervals, fetal growth restriction and preterm birth, child nutrition and infection, and environmental factors. They found that a large number of stunting cases were attributable to being born small for gestational age at term (10.8 million, 24% of cases), followed by poor sanitation (7.2 million, 16% of cases) and diarrhoea (5.8 million, 13% of cases).^11^ In a systematic review, Mosites *et al* calculated the population-attributable fraction of stunting for five categories of risk factors: low birth weight, insufficient diet, enteric dysfunction, infection and toxin exposure. They estimated that 25% of global stunting could be attributed to child diarrhoea incidence. In Africa, the largest number of stunting cases was attributable to low dietary diversity, low feeding frequency, or both (30%).^12^ Another study using population-based surveys from 54 countries estimated that mothers in the lowest category of height (<145 cm) were 2.1 times more likely to have stunted children than mothers in the highest category (≥160 cm).^8^

The key risk factors for linear growth faltering that we identified were similar to those identified in these studies despite several differences in methodology, including prospective cohorts rather than cross-sectional design, examination of both direct and indirect associations through path analysis, and examination of LAZ as a continuous variable rather than stunting cases. By analysing associations with LAZ, we were able to estimate the difference in LAZ with each SD change in predictor variables. Each SD difference in maternal height (5–6 cm) was associated with 0.25 difference in LGAZ at birth (0.5 cm) and a 0.2–0.3 difference in child 18-month LAZ (0.5–0.8 cm). Each SD difference in LGAZ at birth (1.9 cm) was associated with about a 0.5 difference in child 18-month LAZ (1.4 cm). Each SD difference in gestational age at birth (1.8 weeks) was associated with a 0.13–0.17 difference in 18-month LAZ (0.3–0.5 cm). The coefficient sizes for the other key risk factors (maternal BMI, improved water source, dietary diversity, child diarrhoea and Hb) were smaller in magnitude (0.06–0.12).

Despite the examination of 42 environmental, maternal, caregiving and child factors measured longitudinally throughout most of the first 1000 days, our models accounted for a relatively small proportion of the variance in 18-month LAZ (17%–37%), and a large proportion of that was due to maternal height (10%–14%). Factors that were not associated with LAZ were indicators of maternal iron status, illness and inflammation during pregnancy, maternal stress, depression and cognition, exclusive breast feeding during 6 months post partum, infant feeding frequency and child fever, malaria and acute respiratory infections. Variance that remained unaccounted for could be due to unmeasured factors, such as enteric dysfunction, asymptomatic infections, microbiome composition, maternal HIV status or aspects of the diet that were not assessed. It is also possible that population-level factors, such as community sanitation or access to healthcare, may be stronger drivers of growth stunting compared with individual or household-level factors.

Our finding that the pooled analysis accounted for more variance in LAZ (25%) compared with separate analyses by cohort (11%–21%) also supports this argument, although this difference is not very large. Excluding the variance accounted for by maternal height, the pooled analysis accounted for two to three times the variance in LAZ (16%) compared with the separate cohort models (5%–8%). This may be due to the lack of inclusion of a cohort experiencing healthy growth. All four iLiNS cohorts experienced growth faltering, although the cohorts in Malawi and Burkina Faso had faltered 1.5–1.9 SD below the WHO median on average, compared with the Ghanaian children whose mean LAZ was only 0.8 SD below the WHO median by age 18 months. Another potential reason is that we did not measure community-level or social factors that may contribute to children’s growth faltering at a population level. For example, several studies have found that community-level sanitation coverage predicts child growth more strongly than household-level sanitation.^29 30^

Strengths of the study were the large number of children in each cohort, the large number of variables examined and the availability of data from four cohorts of children in three African countries, with many variables harmonised across cohorts, enabling the examination of the same factors and pathways across contexts. Another strength was that the cohorts were enrolled either during pregnancy or at 6 or 9 months post partum and followed prospectively through 18 months post partum, allowing these risk factors to be examined throughout a large portion of the first 1000 days after conception. The prospective design allowed associations in the path model to be drawn from variables measured at earlier time points (eg, pregnancy, birth) to variables measured at later time points (eg, 6 months, 18 months), reducing the risk of reverse causality. However, this does not prevent the possibility of residual confounding, that is, unmeasured variables accounting for observed associations, therefore we are not able to establish causality. Another limitation was that we were not able to explore certain risk factors, such as asymptomatic infections or enteric dysfunction. We reported multiple comparisons; therefore, may have found some false positive associations due to chance. However, we reduced this possibility by reporting p values corrected for multiple comparisons and by basing our primary conclusions on findings that were replicated in more than one cohort. These samples may not be representative of their populations due to their participation in the nutritional supplementation trials. However, any bias introduced by the supplementation would be expected to decrease rather than increase the strength of the associations.^31^ Therefore, our conclusions regarding consistent significant associations would remain the same.

## Conclusion

Our findings may help to inform the design of interventions to pursue United Nations’ Sustainable Development Goal 2, to end hunger and achieve improved nutrition by 2030, with the reduction of stunting prevalence as a key indicator of progress towards this goal. The evidence we present shows that child linear growth status is closely linked to higher maternal height and BMI, fetal growth, full-term birth, improved household water supply, higher child dietary diversity, Hb during infancy and reduced childhood diarrhoea incidence, but we did not find associations with maternal iron status, illness and inflammation during pregnancy (including maternal HIV infection in the one cohort in which it was measured), maternal stress and depression, exclusive breastfeeding during 6 months post partum, infant feeding frequency or child fever, malaria and acute respiratory infections. Much of the variance in linear growth status remained unaccounted for by the 42 individual-level factors measured in this study, suggesting that interventions targeting other individual-level factors or community-level changes may be needed to achieve substantial progress in stunting reduction. The large amount of unexplained variance also suggests that further research is needed to understand the causes of linear growth faltering in order to design more effective strategies to reach the WHO’s target to achieve a 40% reduction in the number of children under-5 who are stunted by 2025.
