# Supplementary material for: Path analyses of risk factors for linear growth faltering in four prospective cohorts of young children in Ghana, Malawi and Burkina Faso
Source: BMJ Glob Health. 2019 Jan 13;4(1):e001155. doi: 10.1136/bmjgh-2018-001155 (PMC6350712; doi:10.1136/bmjgh-2018-001155)
Supplement: Supplementary data [file bmjgh-2018-001155supp001.pdf]

Supplemental Material for the Manuscript "Path Analyses of Risk Factors for Linear Growth Faltering in Four Prospective Cohorts of Young Children in Ghana, Malawi, and Burkina Faso"

Supplemental Methods

iLiNS Project Trial Designs

In the iLiNS-ZINC trial in Burkina Faso, the intervention group received 20 g LNSs/d containing different amounts of zinc and weekly diarrhea and malaria surveillance and treatment, while the control group received no contact between enrollment (9 months) and 18 months. In the iLiNS-DOSE trial, the intervention group received different daily doses of LNSs (10-40 g/day) and weekly diarrhea and malaria surveillance and referral, while the control group only received weekly diarrhea and malaria surveillance and referral from 6 to 18 months. In the two iLiNS-DYAD trials, the intervention group received maternal LNSs (20 g/d) during pregnancy and until 6 months postpartum and child LNS (20 g/d) from age 6 to 18 months. The first control group received a daily maternal multiple micronutrient (MMN) tablet until 6 months postpartum and the second control group received a daily iron and folic acid (IFA) tablet until delivery and a placebo tablet from delivery to 6 months postpartum. Children in the two control groups did not receive LNSs. All groups received bi-weekly morbidity surveillance and referral during pregnancy and weekly morbidity surveillance and referral for the infant after birth.

Ethical approval for the iLiNS-ZINC study procedures was obtained from the Institutional Review Board of the University of California Davis and the Comité d'Ethique Institutionnel du Centre Muraz, Bobo Dioulasso. The study was registered with the U.S. National Institute of Health as a clinical trial (www.ClinicalTrials.gov; NCT00944281). Ethical approval for the iLiNS-DOSE and iLiNS-DYAD-M study procedures was obtained from the University of Malawi, College of Medicine Research and Ethics Committee and the Ethics Committee at

Tampere University Hospital District, Finland. These studies were also registered with the U.S. National Institute of Health as a clinical trial (www.ClinicalTrials.gov; NCT00945698 and NCT01239693). Ethical approval for the iLiNS-DYAD-G study procedures was obtained from the Ethics Committees at the University of California, Davis, the Ghana Health Service, and the University of Ghana Noguchi Memorial Institute for Medical Research (www.ClinicalTrials.gov; NCT00970866). All participants provided written informed consent, by signature or thumb-print.

Participants

Baseline characteristics of the sample assessed for 18-month length compared to those who enrolled but were not assessed have been published previously.[1-5] In cohorts A, C and D, several differences were significant, but the absolute differences were small. In Cohort A, participants lost to follow-up were older (27.8 compared with 26.6 y) and had lower mean gestational age (15.5 compared with 16.2 wk) at enrollment. In Cohort C, child baseline WAZ and WLZ for those measured were -0.74 and 0.29 compared with -0.96 and 0.06, respectively, for those not assessed for 18-month length. In cohort D, mothers of children who completed the study were slightly older than those of non-completers (27.0 vs. 26.2 y). These differences are likely of little practical significance and it is likely that the sample included in these analyses is representative of the full sample in each cohort. In Cohort B, a large proportion of the mothers of children who did not participate in developmental assessment (70%) had enrolled at the public hospital in Mangochi, which was a more transient area compared to the other two sites at Malindi and Lungwena. Compared to those who were lost to follow-up, enrolled mothers whose children did not participate were significantly younger and had higher body mass index (BMI) and lower Hb at baseline, and a greater proportion were primiparous.

Supplemental Table 1. Description of Variables

	Cohort A: DYAD-Ghana	Cohort B: DYAD-Malawi	Cohort C: DOSE-Malawi	Cohort D: ZINC-Burkina Faso
Environmental Factors				
Poverty				
Asset Index	For each trial, we used principal components analysis to construct an asset index based on ownership of a set of assets and household characteristics.[6]			
Household Food Insecurity Access Scale	The household food insecurity access (HFIA) scale was based on a set of questions that captures perceptions and reported experiences of three domains of food insecurity: (1) anxiety about the household food supply; (2) insufficient quality; and (3) insufficient food intake and its physical consequences.[7] In each trial, we calculated the z-score by month of collection, in order to adjust for seasonal changes in food security.			
Distance to Nearest Market	Same as cohorts C and D	-	Coordinates of household and market locations were collected by handheld Global Positioning Systems (GPS). The shortest route along the road network (in meters) from each household to the nearest market was calculated using ArcMap (Environmental Systems Research Institute, Inc., Redlands, CA).	
Access to Education				
Maternal Education	Years of completed formal education			Completed any formal education
Paternal Education	Years of completed formal education			Completed any formal education
Water, Sanitation, and Hygiene				
Unimproved water source	Unimproved water: unprotected well, surface water (river, pond, lake, etc.)<br>Improved water: indoor or outdoor plumbing or pipe, water vendor, borehole, protected well	Unimproved water: unprotected well, lake, river<br>Improved water: piped water, borehole, protected well		Unimproved water: uncovered well, surface water (river, etc.)<br>Improved water: piped water inside or outside the household

				compound, covered well, pump
Unimproved toilet facility	Unimproved toilet: pan/bucket, no toilet facility (bush, beach, etc.)	Unimproved toilet: regular pit latrine, no toilet facility		Unimproved toilet: unimproved latrine, no toilet facility (nature)
	Improved toilet: public or private flush toilet (water closet), private pit latrine	Improved toilet: water closet, improved pit latrine		Improved toilet: water closet with running water, improved latrine
Maternal Factors				
Maternal Age	Age in years at baseline			
Maternal Nutritional Status				
Maternal Height	Height in cm at baseline			
Maternal Body Mass Index (BMI; kg/m^2)	Pre-pregnancy BMI was estimated based on BMI and gestational age at enrollment		Calculated as kg/m^2 at enrollment	
Maternal Baseline[c] Hb (g/dL)	Determined from venous blood samples using Hemocue (Hemocue AG, Wetzikon, Switzerland)	Determined using on-site cuvette readers (HemoCue AB; Angelholm).	-	-
Maternal Baseline[c] ZPP (µmol/mol heme)	Determined from venous blood samples using Hematofluorometer (Aviv Biomedical Co. NJ, USA), after red blood cells were washed three times with normal saline.		-	-
Maternal Baseline[c] sTfR (mg/L)	-	Determined from venous blood samples by immunoturbidimetry on the Cobas Integra 400 system autoanalyzer (F. Hoffmann-La Roche Ltd, Basel, Switzerland).	-	-

Maternal Illness and Inflammation				
Maternal HIV Positive	-	Determined using a whole-blood antibody rapid test (Alere Determine HIV-1/2; Alere Medical Co, Ltd.). If the result was positive, the test was repeated by using another whole-blood antibody rapid test (Uni-Gold HIV; Trinity Biotech plc).	-	-
Maternal Malaria at Baseline[c]: Positive RDT	Determined using Malaria Rapid Diagnostic Test (Clearview Malaria Combo, Vision Biotech, South Africa)	Determined using Malaria Rapid Diagnostic Test (Clearview Malaria Combo; British Biocell International Ltd.)	-	-
Maternal Baseline[c] AGP (g/L)	Determined using a Cobas Integra 400 plus Automatic Analyzer (Roche Diagnostic Corp., Indianapolis, IN, USA).	Determined using a Cobas Integra 400 system autoanalyzer (F. Hoffmann-La Roche Ltd, Basel, Switzerland)	-	-
Maternal AGP 36 wk gestation			-	-
Maternal Elevated AGP	>=1 g/L at both time points		-	-
Maternal Stress				
Maternal Baseline[c] Basal Cortisol (nmol/L)	Saliva was collected after fasting for 30 minutes using an inert polymer swab (Salimetrics Oral Swab). Samples were measured in duplicate using a high sensitivity ELISA test kit (Salimetrics, Carlsbad, CA). We calculated the residual of basal cortisol (nmol/L, log-transformed) predicted by time of day the saliva sample was taken, time since waking, and		-	-
Maternal Basal Cortisol (nmol/L) at 28 wk gestation			-	-
Maternal Basal Cortisol (nmol/L) at 36 wk gestation			-	-

	time since last meal, in order to adjust for these factors.			
Mean Maternal Basal Cortisol across time points	Calculated as the mean of all available time points for each participant.		-	-
Maternal Baseline Perceived Stress Scale (PSS) score	-	Assessed using the Perceived Stress Scale[8 9]	-	-
Maternal PSS score at 28 wk gestation	-		-	-
Maternal PSS score at 36 wk gestation	-		-	-
Mean Maternal PSS score across time points	-	Calculated as the mean of all available time points for each participant	-	-
Maternal Depression 6 mo pp				
Maternal Depression Score	Assessed using the Edinburgh Post-natal Depression Scale (EPDS)[10]	Assessed using the Self-Reporting Questionnaire (SRQ)[11 12]	-	-
Maternal Cognition 6 mo pp				
Maternal Cognitive Z-Score	-	Calculated as the mean z-score across five cognitive tests z-scores: digit span forward and backward, verbal fluency for food and people's names, and mental rotation test.	-	-
Maternal Functional Health Literacy Score	-	This test measures the mother's understanding of health messages written in words and pictures, such as medication instructions, health cards, and growth charts. The score is	-	-

	the number of questions answered correctly out of a maximum 36 points.		
Child Factors			
Firstborn	Children of primiparous mothers were considered firstborn.	-	Children were considered firstborn if there were no older siblings in the household roster.
Gestational age at birth	Gestational age at enrolment was mainly determined by ultrasound.	-	-
Length for gestational age z-score at birth	Length for gestational age z-score at birth was calculated based on the INTERGROWTH-21st norms [13]		
Weight for gestational age z-score at birth	Weight for gestational age z-score at birth was calculated based on the INTERGROWTH-21st norms [13]		
Child Hb/Iron Status			
Child 6 or 9-mo Hb (g/dL)	Hb at 6 months was determined using on-site cuvette readers (HemoCue AB; Angelholm).		Hb at 9 months was determined using on-site cuvette readers (HemoCue AB; Angelholm).
Change in Hb 6 or 9 to 18 mo	Calculated by subtracting Hb at 6 months from Hb at 18 months	-	Calculated by subtracting Hb at 9 months from Hb at 18 months
Child 6 or 9-mo ZPP (µmol/mol heme)	ZPP at 6 months determined using a hematofluorometer (206D, AVIV Biomedical Inc., Lakewood, NJ, USA).		ZPP at 9 months was determined using a hematofluorometer (206D, AVIV Biomedical Inc., Lakewood, NJ, USA).
Change in ZPP 6 or 9 to 18 mo	Calculated by subtracting ZPP at 6 months from ZPP at 18 months		-
Child Illness and Inflammation			
Child 6-mo AGP (g/L)	-	-	-

Child 18-mo AGP (g/L)	-	Determined using a Cobas Integra 400 system autoanalyzer (F. Hoffmann-La Roche Ltd, Basel, Switzerland)	-	-
Child Elevated AGP	-	>=1 g/L at both time points	-	-
Highest Quartile Diarrhea Prevalence (>=3.5% of days)	Diarrhea was defined as caregiver report of three or more loose or liquid stools per 24 hours. Diarrhea prevalence was calculated as the proportion of days on which the child experienced diarrhea among all days of observation for that child, excluding children with < half (273) days of the 18-month observation period.		Defined and calculated in the same way as the other cohorts, excluding children with < 90 days of the 12-month observation period.	Defined and calculated in the same way as the other cohorts, excluding children with < 30 days of the 9-month observation period.
Diarrhea Incidence (episodes per year)	-	-	An episode of diarrhea was defined as the period starting the day the child first had diarrhea following a diarrhea-free period of 2 days, and ending on the last day the child had diarrhea that was followed by ≥2 days without diarrhea.	
Highest Quartile Fever Prevalence (>=2.5% of days)	Fever was defined as any reported fever by the caregiver in the absence of respiratory symptoms (cough, rapid breathing, difficult breathing or nasal discharge) or diarrhea. Fever prevalence was calculated as the proportion of days on which a child experienced fever among all days of observations for that child.	Fever was defined as any elevated measured auricular temperature (>37.5°C). Fever prevalence was calculated as the proportion of days on which the child experienced fever among all days of observation for that child, excluding children with < 90 days of observation. -		Fever was defined as any reported fever by the caregiver or any elevated measured auricular temperature (>37.5°C). Fever prevalence was calculated as the proportion of days on which the child experienced fever among all days of observation for that child, excluding children with < 30 days of observation.

Malaria or Undefined Fever Incidence (episodes per year)	-	-	Fever episodes in the absence of diarrhea and respiratory symptoms, with or without other symptoms, were categorized as 'undefined fever'. Although we did not routinely test for malaria during home visits, cases of 'undefined fever' could be classified as suspected malaria cases according to the Integrated Management of Childhood Illnesses (IMCI) classification for high malaria risk areas.	Any reported or confirmed fever during the 24 h preceding the morbidity visit, associated with a positive RDT was defined as malaria. A child was considered at risk for malaria if the child did not receive any antimalarial treatment within the previous 21 days. Malaria incidence was defined as the number of new episodes of malaria per 100 child-days at risk. This was converted to episodes per 365 child days for Table 1, for comparability with Malawi, but episodes per 100 days was used in the analysis.
ARI Incidence (episodes per year)	If diarrhea was absent but the caregiver reported any respiratory symptoms (cough, nasal obstruction, wheezing, severe difficulty breathing, rapid breathing, or nasal discharge), a diagnosis of ARI was made.			Acute respiratory illness (ARI) was defined as any episode in which the caregiver reported cough with respiratory difficulties (wheezing/ stridor or chest in-drawing) or with a purulent nasal discharge.

Child Appetite				
Child % of days reported poor appetite	Proportion of days on which a child was reported to have poor appetite among all days of observations for that child.		-	Same as Cohorts A and B
Child Stress				
Child 6-mo Basal Cortisol (nmol/L)	-	Saliva samples were collected upon arrival at the clinic. Samples were analyzed using ELISA kit (Salimetrics, Carlsbad, CA).We calculated the residual of basal cortisol (nmol/L, log-transformed) predicted by time of day the saliva sample was taken, time since waking, and time since last meal, in order to adjust for these factors.	-	-
Child 12-mo Basal Cortisol (nmol/L)	-		-	-
Child 18-mo Basal Cortisol (nmol/L)	-		-	-
Mean Child Basal Cortisol across time points	-	Calculated as the mean of all available time points for each participant.	-	-
Child 18-mo Physical Activity				
Accelerometer Mean Vector Magnitude	-	Physical activity was measured over 1 week with hip-worn ActiGraph GT3X+ accelerometer (Pensacola, FL, USA). It recorded accelerations in three different axes: vertical, antero-posterior and medio-lateral. We combined the data from these axes as vector magnitude (VM) counts/15s, which we calculated by taking a square root of the sum of squared activity counts of each axis. We averaged the daily means of VM counts after excluding night		-

		time and strings of ≥20 min of zeroes. We only included participants with minimum of 4 days of ≥6 hours of data.[14]		
Mean % of time spent in moderate to vigorous physical activity	-	We calculated the mean daily proportion of time spend in moderate-to-vigorous activity by vertical axis according to the cut-point >=419 counts/15s.[15]		-
Caregiving Factors				
Infant Feeding				
Exclusive Breastfeeding First 6 mo	Caregiver reported feeding the child no foods or liquids other than breastmilk in monthly 24-hr dietary recalls from age 1 to 5 months.		-	-
9-mo 24-hr feeding frequency	-	Caregiver recalls of the number of times child was fed solid or semi-solid food yesterday.[16]	-	Same as Cohort B
12-mo 24-hr feeding frequency	-		-	-
15-mo 24-hr feeding frequency	-		-	-
18-mo 24-hr feeding frequency	-		-	Same as Cohort B
Mean feeding frequency across time points	-	Calculated as the mean of all available time points for each participant.	-	Calculated as the mean of all available time points for each participant.
9-mo 24-hr dietary diversity	-	-	-	Calculated as the sum of 7 food groups consumed yesterday, with food groups defined as in WHO (2010). World Health Organization, 2010 #6797}
18-mo 24-hr dietary diversity	-	-	-	
Mean 24-hr dietary diversity	-	-	-	Calculated as the mean of all available time points for each participant.
9-mo 7-day dietary diversity score	Sum of whether 10 nutrient-rich food groups were fed to child at least 3 days in past week			Sum of whether 10 nutrient-rich food groups plus 1 category
12-mo 7-day dietary diversity score				
15-mo 7-day dietary diversity score				

18-mo 7-day dietary diversity score	for starchy foods were fed to child at least 3 days in past week.
Mean 7-day dietary diversity score	Calculated as the mean of all available time points for each participant.
Nurturing and Stimulation	
18-mo FCI Variety of Play Materials	Using the Family Care Indicators (FCI) interview,[17 18] the mother reported whether seven types of play materials were available to the child in the home (e.g. toys for pretending, toys for stacking or building).
18-mo FCI Activities with Caregivers	For each of six activities, mothers reported whether the child's mother, father, and any other adult had engaged in that activity with the child in the past three days (e.g. told stories, sang songs). We calculated the total score as the sum of these 18 item scores (6 activities for each of the three categories of potential caregivers).

Supplemental Table 2. Summary Statistics for All Variables Examined in Each Cohort

	Cohort A: DYAD-Ghana		Cohort B: DYAD-Malawi		Cohort C: DOSE-Malawi		Cohort D: ZINC-Burkina Faso	
	n	Mean (SD) or Median (SD) or n (%)	n	Mean (SD) or Median (SD) or n (%)	n	Mean (SD) or Median (SD) or n (%)	n	Mean (SD) or Median (SD) or n (%)
Intergenerational Factors								
Maternal Height (cm): mean (SD)	1023	159 (6)	681	156 (6)	1502	156 (6)	2617	162 (6)
Environmental Factors								
Poverty								
Household Asset Index: median (SD)	1037	0.02 (1)	675	-0.33 (1)	1277	-0.4 (1)	2608	-0.13 (1)
Household Food Insecurity Access Scale: median (SD)	1035	0 (4)	675	4 (4)	1245	5 (6)	2603	1 (4)
Distance to Nearest Market (m): median (SD)	1033	1235 (1851)	-	-	1504	2361 (2763)	2559	828 (1801)
Access to Education								
Paternal Education (y): mean (SD)	879	9 (3)	659	5 (4)	1438	6 (4)	-	-
Father no education: n (%)	-	-	-	-	-	-	2607	777 (30 %)
Maternal Education (y): mean (SD)	1039	8 (4)	680	4 (3)	1470	5 (4)	-	-
Mother no education: n (%)	-	-	-	-	-	-	2608	1555 (60 %)
Water and Sanitation								
Unimproved water source: n (%)	1037	65 (6 %)	683	61 (9 %)	1295	104 (8 %)	2607	1914 (73 %)
Unimproved toilet facility: n (%)	1037	29 (3 %)	683	617 (90 %)	1294	1266 (98 %)	2607	2548 (98 %)
Maternal Factors								
Maternal Age (y): mean (SD)	1039	27 (5)	684	25 (6)	1463	26 (6)	2600	27 (7)
Maternal Nutritional Status								
Maternal Body Mass Index (kg/m2): mean (SD)	1022	24 (4)	672	21 (3)	1495	22 (3)	2617	21 (2)
Maternal Baseline[a] Hb (g/dL): mean (SD)	1039	11 (1)	683	11 (2)	-	-	-	-
Maternal Baseline[a] ZPP (µmol/mol heme): median (SD)	1037	37 (31.2)	683	42 (40)	-	-	-	-
Maternal Baseline[a] TfR (mg/L): median (SD)	-	-	671	4 (3)	-	-	-	-

Maternal Illness and Inflammation								
Maternal HIV Positive: n (%)	-	-	681	82 (12 %)	-	-	-	-
Maternal Malaria at Baseline[a]: Positive RDT: n (%)	1039	98 (9 %)	681	155 (23 %)	-	-	-	-
Maternal Baseline[a] AGP (g/L): median (SD)	1018	0.6 (0.2)	671	0.7 (0.2)	-	-	-	-
Maternal AGP 36 wk gestation: median (SD)	887	0.4 (0.2)	571	0.5 (0.2)	-	-	-	-
Maternal Elevated AGP (>=1 g/L) at both time points: n (%)	871	4 (0 %)	566	11 (2 %)	-	-	-	-
Maternal Stress								
Maternal Baseline[a] Basal Cortisol (nmol/L): median (SD)	718	4.3 (2.7)	665	5 (4)	-	-	-	-
Maternal Basal Cortisol (nmol/L) at 28 wk gestation: median (SD)	684	5.8 (3.4)	486	5 (4)	-	-	-	-
Maternal Basal Cortisol (nmol/L) at 36 wk gestation: median (SD)	594	7.5 (3)	562	8 (3)	-	-	-	-
Mean Maternal Basal Cortisol across time points: median (SD)	-	-	681	6 (3)	-	-	-	-
Maternal Baseline Perceived Stress Scale (PSS) score: mean (SD)	-	-	639	14 (5)	-	-	-	-
Maternal PSS score[b] at 28 wk gestation: mean (SD)	-	-	509	15 (6)	-	-	-	-
Maternal PSS score[b] at 36 wk gestation: mean (SD)	-	-	561	14 (6)	-	-	-	-
Mean Maternal PSS score[b] across time points: mean (SD)	-	-	682	14 (4)	-	-	-	-
Maternal Depression 6 mo pp								
Maternal Depression Score: mean (SD)	1003	5[c] (5)	618	2[d] (3)	-	-	-	-
Maternal Cognition 6 mo pp								
Maternal Cognitive Z-Score[e]: mean (SD)	-	-	641	0 (1)	-	-	-	-
Maternal Functional Health Literacy Score[f]: mean (SD)	-	-	644	16 (4)	-	-	-	-
Child Factors								
Firstborn: n (%)	1039	344 (33 %)	682	146 (21 %)	-	-	2117	502 (19 %)
Gestational age at birth (weeks): mean (SD)	1039	39 (2)	684	39 (2)	-	-	-	-

Length for gestational age z-score at birth: mean (SD)	979	-0.5 (0.9)	604	-1.0 (1.0)	-	-	-	-
Weight for gestational age z-score at birth: mean (SD)	983	-0.5 (1.0)	612	-0.4 (1.0)	-	-	-	-
Child Hb/Iron Status								
Child 6 or 9-mo Hb (g/dL): mean (SD)	876	11[g] (1)	648	10[g] (2)	1493	10[g] (2)	2619	9[h] (2)
Change in Hb 6 or 9 to 18 mo: mean (SD)	839	-0.1[g] (1.1)	624	1[g] (2)	-	-	2614	1[h] (2)
Child 6 or 9-mo ZPP (Δµmol/mol heme): median (SD)	845	58 (47)	573	78 (72)	1438	83 (85)	2612	171 (114)
Change in ZPP 6 to 18 mo: mean (SD)	798	-1 (50)	513	-16 (70)	1019	-8 (95)	-	-
Child Illness and Inflammation								
Child 6-mo AGP (g/L): median (SD)	-	-	532	1.2 (0.4)	-	-	-	-
Child 18-mo AGP (g/L): median (SD)	-	-	608	1.3 (0.6)	-	-	-	-
Child Elevated AGP (>=1 g/L) at both 6 and 18 mo: n (%)	-	-	654	361 (55 %)	-	-	-	-
Highest Quartile Diarrhea Prevalence (>=3.5% of days): n (%)	1016	260 (26 %)	508	143 (22 %)	1458	2.1[i] (4.5)	1954	2.2[i] (3.2)
Diarrhea Incidence (episodes per year): median (SD)	-	-	-	-	1457	1.2 (2.2)	1954	3.0 (3.7)
Highest Quartile Fever Prevalence (>=2.5% of days): n (%)	1016	233 (23 %)	546	105 (16 %)	1458	6.6[i] (6.9)	1954	2.9[i] (2.8)
Malaria or Undefined Fever Incidence (episodes per year): median (SD)	-	-	-	-	1457	1.2[j] (1.6)	1954	1.7[k] (2.4)
ARI Incidence (episodes per year): median (SD)	1016	2.8 (2)	651	2.3 (1.8)	-	-	1954	1342[l] (69 %)
Child Appetite								
Prevalence of reported poor appetite (% of days): median (SD)	1016	3.5 (6.3)	651	2.0 (3.5)	-	-	1954	2.6 (3.6)
Child Stress								
Child 6-mo Basal Cortisol (nmol/L): median (SD)	-	-	618	4 (5)	-	-	-	-
Child 12-mo Basal Cortisol (nmol/L): median (SD)	-	-	603	4 (6)	-	-	-	-
Child 18-mo Basal Cortisol (nmol/L): median (SD)	-	-	648	3 (5)	-	-	-	-
Mean Child Basal Cortisol across time points: median (SD)	-	-	679	4 (3)	-	-	-	-
Child 18-mo Activity								

Accelerometer Mean Vector Magnitude: mean (SD)	-	-	578	302 (57)	1073	305 (65)	-	-
Mean % of time spent in moderate to vigorous physical activity: mean (SD)	-	-	578	11 (4)	1073	12 (4)	-	-
Caregiving Factors								
Infant Feeding								
Exclusive Breastfeeding First 6 mo: n (%)	807	428 (53 %)	525	125 (24 %)	-	-	-	-
9-mo 24-hr feeding frequency: mean (SD)	-	-	543	2 (1)	-	-	2374	2 (1)
12-mo 24-hr feeding frequency: mean (SD)	-	-	575	2 (1)	-	-	-	-
15-mo 24-hr feeding frequency: mean (SD)	-	-	599	3 (1)	-	-	-	-
18-mo 24-hr feeding frequency: mean (SD)	-	-	621	3 (1)	-	-	2613	3 (1)
Mean feeding frequency across time points: mean (SD)	-	-	671	2 (1)	-	-	2619	3 (1)
9-mo 24-hr dietary diversity: mean (SD)	-	-	-	-	-	-	2618	2[m] (1)
18-mo 24-hr dietary diversity: mean (SD)	-	-	-	-	-	-	2614	3[m] (1)
Mean 24-hr dietary diversity: mean (SD)	-	-	-	-	-	-	2619	3[m] (1)
9-mo 7-day dietary diversity score: mean (SD)	978	3 (2)	516	2 (1)	1162	3 (2)	2558	2[m] (1)
12-mo 7-day dietary diversity score: mean (SD)	1007	3 (2)	536	3 (1)	1100	3 (2)	-	-
15-mo 7-day dietary diversity score: mean (SD)	984	3 (2)	562	3 (1)	1095	3 (1)	-	-
18-mo 7-day dietary diversity score: mean (SD)	1026	4 (2)	591	3 (1)	1281	3 (2)	2569	3[m] (1)
Mean 7-day dietary diversity score: mean (SD)	1039	3 (1)	670	2 (1)	1504	2 (1)	2617	3[m] (1)
Nurturing and Stimulation								
18-mo FCI Variety of Play Materials: mean (SD)	989	3 (1)	668	3 (1)	1350	4 (1)	1118	3 (1)
18-mo FCI Activities with Caregivers: mean (SD)	989	5 (3)	668	3 (3)	1350	3 (2)	1118	8 (3)

[a]Baseline measures were collected at < 20 wk gestation

[b]Out of Maximum 40 points

[c]Score on the Edinburgh Post-Natal Depression Scale, out of maximum 30 points

[d]Score on the Self-Reporting Questionnaire, out of maximum 20 points

[e]Mean z-score across five cognitive tests z-scores: digit span forward and backward, verbal fluency for food and people's names, and mental rotation test.

[f]Out of Maximum 36 points

[g]6 months

[h]9 months

[i]median(SD)

[j]Fever in the absence of diarrhea and respiratory symptoms, which can be classified as suspected malaria cases according to the Integrated Management of Childhood Illnesses (IMCI) classification for high malaria risk areas.
[k]Malaria incidence
[l]Child experienced ARI during the surveillance period: n (%)
[m]Includes starchy staple foods, which were not included in the other three cohorts
Note: Where the distribution shows n(%), the binary variable was used for analysis. Where the distribution shows mean (SD), the continuous variable was used for analysis. Where the distribution shows median (SD), the log-transformed continuous variable was used for analysis

Supplemental Table 3. Variable Selection Results for Cohort A: DYAD-Ghana

	Independent association with 18-month LAZ			Adjusted for other variables in the same category		
	Estimate (SE)	p-value	Decision	Estimate (SE)	p-value	Decision
Intergenerational Factors						
Maternal Height (cm)	0.32 (0.03)	<.0001	retain	0.32 (0.03)	<.0001	retain
Environmental Factors						
Asset Index	0.10 (0.03)	0.00	retain	0.08 (0.04)	0.04	retain
Household Food Insecurity Access Scale	-0.07 (0.03)	0.02	retain	-0.05 (0.04)	0.14	drop
Distance to Nearest Market (m)	-0.01 (0.03)	0.85	drop	-	-	-
Paternal Education (y)	0.09 (0.03)	0.01	retain	0.05 (0.04)	0.13	drop
Maternal Education (y)	0.03 (0.03)	0.39	drop	-	-	-
Unimproved water source	-0.07 (0.13)	0.58	drop	-	-	-
Unimproved toilet facility	-0.13 (0.19)	0.48	drop	-	-	-
Maternal Factors						
Maternal Age (y)	0.03 (0.03)	0.33	drop	-	-	-
Maternal Body Mass Index (kg/m2)	0.07 (0.03)	0.03	retain	0.05 (0.03)	0.10	drop
Maternal Malaria at Baseline: Positive RDT	-0.13 (0.11)	0.23	drop	-	-	-
Maternal Baseline Hb Concentration	0.11 (0.03)	0.00	retain	0.1 (0.03)	0.00	retain
Maternal Baseline ZPP Concentration (µmol/mol heme)	0 (0.03)	0.89	drop	-	-	-
Maternal Baseline AGP	-0.03 (0.03)	0.38	drop	-	-	-
Maternal AGP 36 wk gestation	-0.05 (0.03)	0.14	drop	-	-	-
Maternal Elevated AGP (>=1) at both Baseline and 36 wk gestation	-0.6 (0.5)	0.23	drop	-	-	-
Maternal Baseline Basal Cortisol	-0.03 (0.04)	0.46	drop	-	-	-
Maternal Basal Cortisol at 28 wk gestation	-0.06 (0.04)	0.16	drop	-	-	-
Maternal Basal Cortisol at 36 wk gestation	-0.01 (0.04)	0.88	drop	-	-	-
Mean Maternal Basal Cortisol across the three time points	-0.05 (0.04)	0.20	drop	-	-	-
Maternal Depression Score	0.02 (0.03)	0.48	drop	-	-	-
Child Factors						
Gestational Age at Birth	0.13 (0.03)	<.0001	retain	0.13 (0.03)	<.0001	retain
Length for Gestational Age (LGAZ) at Birth	0.57 (0.03)	<.0001	retain	0.57 (0.03)	<.0001	retain
Weight for Gestational Age (WGAZ) at Birth	0.33 (0.03)	<.0001	drop[1]	-	-	-
Firstborn	0.11 (0.07)	0.10	drop	-	-	-
Child 6-month Hb Concentration (g/L)	-0.02 (0.04)	0.69	drop	-	-	-
Change in Hb 6 to 18 months	0 (0.04)	0.91	drop	-	-	-
Child 6-month ZPP Concentration	0.05 (0.04)	0.18	drop	-	-	-

Change in ZPP 6 to 18 months	0.04 (0.04)	0.25	drop	-	-	-
Highest Quartile Diarrhea Prevalence (>=3.5% of days)	0 (0.07)	0.99	drop	-	-	-
Highest Quartile Fever Prevelance (>=2.5% of days)	-0.04 (0.07)	0.57	drop	-	-	-
ARI Prevalence (% of days)	-0.04 (0.03)	0.25	drop	-	-	-
Prevalence of reported poor appetite (% of days)	-0.06 (0.03)	0.04	retain	-0.06 (0.03)	0.04	retain
Caregiving Factors						
Exclusive Breastfeeding First 6 mo	0.08 (0.07)	0.27	drop	-	-	-
9-month dietary diversity score	0.04 (0.03)	0.25	drop	-	-	-
12-month dietary diversity score	0.05 (0.03)	0.12	drop	-	-	-
15-month dietary diversity score	0.02 (0.03)	0.48	drop	-	-	-
18-month dietary diversity score	0.05 (0.03)	0.12	drop	-	-	-
Mean dietary diversity score	0.05 (0.03)	0.11	drop	-	-	-
18-Month variety of play materials	0.02 (0.03)	0.54	drop	-	-	-
18-Month activities with caregivers	0.03 (0.03)	0.28	drop	-	-	-

[1]Drop due to collinearity with LGAZ at birth

Supplemental Table 4. Variable Selection Results for Cohort B: DYAD-Malawi

	Independent association with 18-month LAZ			Adjusted for other variables in the same category		
	Estimate (SE)	p-value	Decision	Estimate (SE)	p-value	Decision
Intergenerational factors						
Maternal Height (cm)	0.40 (0.04)	<.0001	retain	0.40 (0.04)	<.0001	retain
Environmental Factors						
Asset Index	0.14 (0.04)	0.00	retain	0.04 (0.06)	0.54	drop
Household Food Insecurity Access Scale	-0.09 (0.04)	0.03	retain	-0.05 (0.05)	0.26	drop
Paternal Education (y)	0.14 (0.04)	0.00	retain	0.08 (0.05)	0.11	retain
Maternal Education (y)	0.11 (0.04)	0.01	retain	0.03 (0.05)	0.53	drop
Unimproved water source	0.06 (0.15)	0.66	drop	-	-	-
Unimproved toilet facility	-0.37 (0.14)	0.01	retain	-0.21 (0.18)	0.24	drop
Maternal Factors						
Maternal Age (y)	-0.01 (0.04)	0.72	drop	-	-	-
Maternal Body Mass Index (kg/m2)	0.09 (0.04)	0.03	retain	0.09 (0.05)	0.07	retain
Maternal HIV Positive	-0.25 (0.13)	0.052	drop	-	-	-
Maternal Malaria at Baseline: Positive RDT	-0.06 (0.1)	0.56	drop	-	-	-
Maternal Baseline Hb Concentration (g/L)	0.03 (0.04)	0.47	drop	-	-	-
Maternal Baseline ZPP Concentration (µmol/mol heme)	-0.01 (0.04)	0.83	drop	-	-	-
Maternal Baseline Serum (?)Tfr	-0.03 (0.04)	0.48	drop	-	-	-
Maternal Baseline AGP	-0.14 (0.04)	0.00	retain	-0.07 (0.05)	0.16	drop
Maternal AGP 36 wk gestation	-0.09 (0.05)	0.0498	retain	-0.03 (0.05)	0.56	drop
Maternal Elevated AGP (>=1) at both Baseline and 36 wk gestation	-0.33 (0.33)	0.32	drop	-	-	-
Maternal Baseline Basal Cortisol	-0.21 (0.11)	0.06	drop	-	-	-
Maternal Basal Cortisol at 28 wk gestation	-0.17 (0.17)	0.32	drop	-	-	-
Maternal Basal Cortisol at 36 wk gestation	0.39 (0.30)	0.25	drop	-	-	-
Mean Maternal Basal Cortisol across the three time points	-0.19 (0.1)	0.052	drop	-	-	-
Maternal Baseline Perceived Stress Scale (PSS) score	0.04 (0.04)	0.32	drop	-	-	-
Maternal PSS score at 28 wk gestation	0.02 (0.05)	0.64	drop	-	-	-
Maternal PSS score at 36 wk gestation	-0.02 (0.05)	0.65	drop	-	-	-
Mean Maternal PSS score across the three time points	0.03 (0.04)	0.53	drop	-	-	-
Maternal Depression Score	0.01 (0.04)	0.75	drop	-	-	-
Maternal Cognitive Score	0.1 (0.04)	0.03	retain	0.06 (0.05)	0.24	drop
Maternal Functional Health Literacy Score	0.09 (0.04)	0.04	retain	0.05 (0.05)	0.33	drop

Child Factors						
Gestational age at birth	0.12 (0.04)	0.00	retain	0.12 (0.04)	0.00	retain
Length for Gestational Age Z-Score (LGAZ) at Birth	0.54 (0.04)	<.0001	retain	0.54 (0.04)	<.0001	retain
Weight for Gestational Age Z-Score (WGAZ) at Birth	0.50 (0.04)	<.0001	drop[1]			
Firstborn	0.05 (0.10)	0.64	drop	-	-	-
Child 6-month Hb Concentration	0.17 (0.05)	0.00	retain	0.15 (0.06)	0.01	retain
Change in Hb 6 to 18 months	0.13 (0.05)	0.01	retain	0.07 (0.06)	0.24	retain[3]
Child 6-month ZPP Concentration	-0.08 (0.06)	0.18	drop	-	-	-
Change in ZPP 6 to 18 months	0.00 (0.06)	0.97	drop	-	-	-
Child 6-month AGP	-0.03 (0.05)	0.45	drop	-	-	-
Child 18-month AGP	-0.18 (0.04)	<.0001	retain	-0.12 (0.05)	0.03	retain
Child Elevated AGP (>=1) at both 6 and 18 months	-0.18 (0.08)	0.03	drop[2]	-	-	-
Highest Quartile Diarrhea Prevalence (>=3.5% of days)	-0.17 (0.1)	0.10	drop	-	-	-
Highest Quartile Fever Prevalence (>=2.5% of days)	-0.13 (0.12)	0.26	drop	-	-	-
ARI Incidence (episodes per year)	0.00 (0.04)	0.91	drop	-	-	-
Prevalence of reported poor appetite (% of days)	-0.04 (0.04)	0.33	drop	-	-	-
Child 6-month Basal Cortisol	0.05 (0.05)	0.29	drop	-	-	-
Child 12-month Basal Cortisol	0.08 (0.05)	0.10	drop	-	-	-
Child 18-month Basal Cortisol	-0.01 (0.04)	0.85	drop	-	-	-
Mean Child Basal Cortisol across the three time points	0.04 (0.04)	0.31	drop	-	-	-
Accelerometer Mean Vector Magnitude	0.16 (0.05)	0.00	retain	0.07 (0.05)	0.15	drop
Mean % of time spent in moderate to vigorous physical activity	0.05 (0.05)	0.32	drop	-	-	-
Caregiving Factors						
Exclusive Breastfeeding First 6 months	-0.24 (0.22)	0.28	drop	-	-	-
9-month feeding frequency yesterday	0.03 (0.04)	0.49	drop	-	-	-
12-month feeding frequency yesterday	-0.03 (0.05)	0.58	drop	-	-	-
15-month feeding frequency yesterday	0.00 (0.05)	0.96	drop	-	-	-
18-month feeding frequency yesterday	-0.04 (0.04)	0.36	drop	-	-	-
Mean feeding frequency across the four time points	0.02 (0.04)	0.72	drop	-	-	-
9-month dietary diversity score	0.05 (0.05)	0.32	drop	-	-	-
12-month dietary diversity score	-0.03 (0.05)	0.54	drop	-	-	-
15-month dietary diversity score	0.07 (0.05)	0.13	drop	-	-	-
18-month dietary diversity score	0.01 (0.05)	0.74	drop	-	-	-
Mean dietary diversity score	0.03 (0.04)	0.44	drop	-	-	-
18-Month variety of play materials	0.11 (0.04)	0.01	retain	0.1 (0.04)	0.02	retain
18-Month activities with caregivers	0.14 (0.04)	0.00	retain	0.12 (0.04)	0.00	retain

[1]Drop due to collinearity with LGAZ at birth

[2]Drop due to collinearity with child 18-month AGP

[3]Retain as a covariate

Supplemental Table 5. Variable Selection Results for Cohort C: DOSE-Malawi

	Independent association with 18-month LAZ			Adjusted for other variables in the same category		
	Estimate (SE)	p-value	Decision	Estimate (SE)	p-value	Decision
Intergenerational Factors						
Maternal Height	0.30 (0.03)	<.0001	retain	0.30 (0.03)	<.0001	retain
Environmental Factors						
Asset Index	0.13 (0.03)	<.0001	retain	0.02 (0.04)	0.55	drop
Household Food Insecurity Access Scale	-0.06 (0.03)	0.045	retain	-0.01 (0.03)	0.80	drop
Distance to Nearest Market	-0.14 (0.03)	<.0001	retain	-0.07 (0.03)	0.04	retain
Paternal Education	0.14 (0.03)	<.0001	retain	0.03 (0.04)	0.48	drop
Maternal Education	0.16 (0.03)	<.0001	retain	0.11 (0.04)	0.00	retain
Unimproved water source	-0.35 (0.11)	0.00	retain	-0.32 (0.12)	0.01	retain
Unimproved toilet facility	-0.30 (0.21)	0.15	drop	-	-	-
Maternal Factors						
Maternal Age	0.00 (0.03)	0.87	drop	-	-	-
Maternal Body Mass Index	0.13 (0.03)	<.0001	retain	0.13 (0.03)	<.0001	retain
Child Factors						
Child 6-month Hb Concentration	0.11 (0.03)	<.0001	retain	0.08 (0.03)	0.01	retain
Child 6-month ZPP Concentration	-0.01 (0.04)	0.80	drop	-	-	-
Change in ZPP 6 to 18 months	0.01 (0.04)	0.71	drop	-	-	-
Diarrhea Prevalence	-0.08 (0.03)	0.01	drop[1]	-	-	-
Diarrhea Incidence	-0.09 (0.03)	0.00	retain	-0.06 (0.03)	0.049	retain
Fever Prevelance	-0.06 (0.03)	0.04	retain	-0.01 (0.03)	0.72	drop
Undefined Fever Incidence	-0.03 (0.03)	0.35	drop	-	-	-
ARI Incidence	0.03 (0.03)	0.36	drop	-	-	-
Accelerometer Mean Vector Magnitude	0.14 (0.03)	<.0001	retain	0.13 (0.03)	<.0001	retain
Mean % of time spent in moderate to vigorous physical activity	-0.04 (0.03)	0.22	drop	-	-	-
Caregiving Factors						
9-month dietary diversity score	0.02 (0.03)	0.61	drop	-	-	-
12-month dietary diversity score	0.01 (0.03)	0.65	drop	-	-	-
15-month dietary diversity score	0.06 (0.03)	0.048	retain	0.06 (0.03)	0.048	retain
18-month dietary diversity score	-0.01 (0.03)	0.82	drop	-	-	-
Mean dietary diversity score	0.05 (0.03)	0.10	drop	-	-	-
18-Month variety of play materials	0.05 (0.03)	0.09	drop	-	-	-
18-Month activities with caregivers	0.06 (0.03)	0.06	drop	-	-	-

[1]drop due to collinearity with diarrhea incidence

Supplemental Table 6. Variable Selection Results for Cohort D: ZINC-Burkina Faso

	Independent association with 18-month LAZ			Adjusted for other variables in the same category		
	Estimate (SE)	p-value	Decision	Estimate (SE)	p-value	Decision
Intergenerational Factors						
Maternal Height (cm)	0.31 (0.02)	<.0001	retain	0.31 (0.02)	<.0001	retain
Environmental Factors						
Asset Index	0.05 (0.02)	0.03	retain	0.03 (0.02)	0.25	drop
Household Food Insecurity Access Scale	0.01 (0.02)	0.66	drop	-	-	-
Distance to Nearest Market (m)	-0.04 (0.02)	0.06	drop	-	-	
Father no education	-0.04 (0.05)	0.44	drop	-	-	-
Mother no education	-0.12 (0.04)	0.00	retain	-0.11 (0.04)	0.01	retain
Unimproved water source	-0.14 (0.05)	0.00	retain	-0.11 (0.05)	0.02	retain
Unimproved toilet facility	-0.01 (0.14)	0.95	drop	-	-	-
Maternal Factors						
Maternal Age (y)	0.05 (0.02)	0.01	retain	0.04 (0.02)	0.07	drop
Maternal Body Mass Index (kg/m2)	0.12 (0.02)	<.0001	retain	0.12 (0.02)	<.0001	retain
Child Factors						
Firstborn	-0.10 (0.05)	0.06	drop	-	-	-
Child 9-month Hb Concentration (g/L)	0.16 (0.03)	<.0001	retain	0.15 (0.03)	<.0001	retain
Change in Hb 9 to 18 months	0.13 (0.03)	<.0001	retain	0.10 (0.03)	<0.001	retain
Child 9-month ZPP Concentration	-0.02 (0.02)	0.39	drop	-	-	-
Diarrhea Prevalence (% of days)	-0.04 (0.02)	0.10	drop	-	-	-
Diarrhea Incidence (episodes per 100 d)	-0.05 (0.02)	0.03	retain	-0.07 (0.02)	0.006	retain
Fever Prevelance (% of days)	0.00 (0.02)	0.85	drop	-	-	-
Malaria incidence (episodes per 100 d)	-0.03 (0.02)	0.14	drop	-	-	-
Child had ARI during surveillance period	0.03 (0.05)	0.59	drop	-	-	-
Prevalence of reported poor appetite (% of days)	-0.01 (0.02)	0.54	drop	-	-	-
Caregiving Factors						
9-month meal frequency in past 24 hours	0.03 (0.02)	0.11	drop	-	-	-
18-month meal frequency in past 24 hours	-0.01 (0.02)	0.68	drop	-	-	-
Mean meal frequency in past 24 hours	0.02 (0.02)	0.45	drop	-	-	-
9-month dietary diversity in past 24 hours	0.08 (0.02)	<.0001	drop[1]	-	-	-
18-month dietary diversity in past 24 hours	0.01 (0.02)	0.69	drop	-	-	-
Mean dietary diversity in past 24 hours	0.06 (0.02)	0.00	drop[1]	-	-	-
9-month dietary diversity in past 7 days	0.10 (0.02)	<.0001	retain	0.11 (0.03)	<0.001	retain
18-month dietary diversity in past 7 days	0.03 (0.02)	0.14	drop	-	-	-

Mean dietary diversity in past 7 days	0.08 (0.02)	<.0001	drop1	-	-	-
18-Month variety of play materials	0.18 (0.03)	<.0001	retain	0.16 (0.03)	<.0001	retain
18-Month activities with caregivers	0.09 (0.03)	0.00	retain	0.05 (0.03)	0.15	drop

[1]Drop due to collinearity with the 9-month dietary diversity in past 7 days

Supplemental Table 7. Pathway Selection Results for Cohort A: DYAD-Ghana

Independent Variable	Potential Mediator	Association between IV and Potential Mediator			Indirect effect in Multiple Mediation Model		
		Estimate (SE)	p-value	Decision	Estimate (SE)	p-value	Decision
Maternal Height							
	Asset Index	0.07 (0.03)	0.02	retain	0.005 (0.003)	0.11	drop
	Maternal Hb < 20 weeks gestation	0.06 (0.03)	0.07	drop	-	-	-
	Gestational Age at Birth	0.03 (0.03)	0.31	drop	-	-	-
	LGAZ at Birth	0.24 (0.03)	<.0001	retain	0.113 (0.015)	<.0001	retain
	Child Poor Appetite Prevalence	-0.04 (0.03)	0.21	drop	-	-	-
Asset Index							
	Maternal Hb < 20 weeks gestation	0.2 (0.03)	<.0001	retain	0.018 (0.007)	0.01	retain
	Gestational Age at Birth	0.1 (0.03)	0.00	retain	0.011 (0.004)	0.01	retain
	LGAZ at Birth	0.04 (0.03)	0.16	drop	-	-	-
	Child Poor Appetite Prevalence	0.06 (0.03)	0.08	drop	-	-	-
Maternal Hb < 20 weeks gestation							
	Gestational Age at Birth	0.08 (0.03)	0.01	retain	0.009 (0.005)	0.06	drop
	LGAZ at Birth	0.03 (0.03)	0.33	drop	-	-	-
	Child Poor Appetite Prevalence	0.08 (0.03)	0.02	retain	-0.005 (0.003)	0.12	drop
Gestational Age at Birth							
	Child Poor Appetite Prevalence	0 (0.03)	0.98	drop	-	-	-
LGAZ at Birth							
	Child Poor Appetite Prevalence	-0.04 (0.03)	0.27	drop	-	-	-

Supplemental Table 8. Pathway Selection Results for Cohort B: DYAD-Malawi

Independent Variable	Potential Mediator	Association between IV and Potential Mediator			Indirect effect in Multiple Mediation Model		
		Estimate (SE)	p-value	Decision	Estimate (SE)	p-value	Decision
Maternal Height (cm)							
	Paternal Education	0.15 (0.04)	0.00	retain	0.015 (0.007)	0.03	drop[1]
	Maternal Body Mass Index (kg/m2)	0.00 (0.04)	0.97	drop	-	-	-
	Gestational age at birth	0.08 (0.04)	0.03	retain	0.002 (0.003)	0.53	drop
	LGAZ at birth	0.25 (0.04)	<.0001	retain	0.109 (0.019)	<0.001	retain
	Child 6-month Hb Concentration (g/L)	0.06 (0.04)	0.13	drop	-	-	-
	Child 18-month AGP	-0.01 (0.04)	0.84	drop	-	-	-
	18-Month variety of play materials	0.04 (0.04)	0.32	drop	-	-	-
	18-Month activities with caregivers	-0.01 (0.04)	0.89	drop	-	-	-
Paternal Education							
	Maternal Body Mass Index (kg/m2)	0.17 (0.04)	<.0001	retain	0.010 (0.007)	0.19	drop
	Gestational age at birth	0.02 (0.04)	0.62	drop	-	-	-
	LGAZ at birth	0.05 (0.05)	0.34	drop	-	-	-
	Child 6-month Hb Concentration (g/L)	0.09 (0.04)	0.02	retain	0.012 (0.007)	0.09	drop
	Child 18-month AGP	-0.08 (0.04)	0.06	drop	-	-	-
	18-Month variety of play materials	0.10 (0.04)	0.01	retain	0.006 (0.006)	0.27	drop
	18-Month activities with caregivers	0.00 (0.04)	0.95	drop	-	-	-
Maternal Body Mass Index (kg/m2)							
	Gestational age at birth	0.00 (0.04)	0.91	drop	-	-	-
	LGAZ at birth	0.13 (0.05)	0.01	retain	0.052 (0.020)	0.01	retain
	Child 6-month Hb Concentration (g/L)	0.02 (0.04)	0.60	drop	-	-	-
	Child 18-month AGP	-0.04 (0.04)	0.35	drop	-	-	-
	18-Month variety of play materials	0.06 (0.04)	0.10	drop	-	-	-
	18-Month activities with caregivers	-0.03 (0.04)	0.43	drop	-	-	-
Gestational age at birth							
	Child 6-month Hb Concentration (g/L)	0.19 (0.04)	<.0001	retain	0.017 (0.008)	0.025	retain
	Child 18-month AGP	0.02 (0.04)	0.65	drop	-	-	-
	18-Month variety of play materials	0.04 (0.04)	0.27	drop	-	-	-
	18-Month activities with caregivers	0.03 (0.04)	0.37	drop	-	-	-
LGAZ at birth							
	Child 6-month Hb Concentration (g/L)	0.05 (0.04)	0.14	drop	-	-	-
	Child 18-month AGP	-0.04 (0.04)	0.25	drop	-	-	-
	18-Month variety of play materials	0.07 (0.04)	0.045	retain	0.005 (0.004)	0.26	drop
	18-Month activities with caregivers	0.08 (0.04)	0.02	retain	0.01 (0.006)	0.09	drop
Child 6-month Hb Concentration (g/L)							
	18-Month variety of play materials	0.09 (0.04)	0.02	retain	0.013 (0.008)	0.104	drop
	18-Month activities with caregivers	-0.05 (0.04)	0.19	drop	-	-	-

Child 18-month AGP						
18-Month variety of play materials	0.01 (0.04)	0.81	drop	-	-	-
18-Month activities with caregivers	0.00 (0.04)	0.95	drop	-	-	-

[1]Drop due to significant interaction between maternal height and paternal education.

Supplemental Table 9. Pathway Selection Results for Cohort C: DOSE-Malawi

Independent Variable	Potential Mediator	Association between IV and Potential Mediator			Indirect effect in Multiple Mediation Model		
		Estimate (SE)	p-value	Decision	Estimate (SE)	p-value	Decision
Maternal height							
	Maternal Education	-0.08 (0.03)	0.001	retain	0.013 (0.006)	0.03	drop[1]
	Distance to nearest market	0.13 (0.03)	<.0001	retain	0.005 (0.003)	0.15	drop
	Unimproved Water Source	-0.25 (0.1)	0.013	retain	0.006 (0.007)	0.37	drop
	Maternal BMI	0.00 (0.03)	0.88	drop	-	-	-
	Child 6-month Hb	0.08 (0.03)	0.00	retain	0.012 (0.005)	0.03	retain
	Diarrhea Incidence	0.05 (0.03)	0.06	drop	-	-	-
	Physical Activity: Mean Vector Magnitude	0.03 (0.03)	0.40	drop	-	-	-
	15-Month Dietary Diversity in Past 7 Days	0.08 (0.03)	0.01	retain	0 (0.002)	0.94	drop
Maternal Education							
	Maternal BMI	0.09 (0.03)	0.00	retain	0.011 (0.005)	0.02	retain
	Child 6-month Hb	0.11 (0.03)	<.0001	retain	0.012 (0.005)	0.01	retain
	Diarrhea Incidence	-0.02 (0.03)	0.41	drop	-	-	-
	Physical Activity: Mean Vector Magnitude	-0.02 (0.03)	0.45	drop	-	-	-
	15-Month Dietary Diversity in Past 7 Days	0.11 (0.03)	0.00	retain	0.003 (0.003)	0.33	drop
Distance to nearest market							
	Maternal BMI	-0.15 (0.03)	<.0001	retain	-0.015 (0.005)	0.00	retain
	Child 6-month Hb	-0.01 (0.03)	0.69	drop	-	-	-
	Diarrhea Incidence	0.10 (0.03)	0.00	retain	-0.007 (0.003)	0.04	retain
	Physical Activity: Mean Vector Magnitude	-0.03 (0.03)	0.31	drop	-	-	-
	15-Month Dietary Diversity in Past 7 Days	0.00 (0.03)	0.92	drop	-	-	-
Unimproved Water Source							
	Maternal BMI	-0.26 (0.1)	0.01	retain	-0.007 (0.004)	0.08	drop
	Child 6-month Hb	-0.17 (0.1)	0.10	drop	-	-	-
	Diarrhea Incidence	0.49 (0.1)	<.0001	retain	-0.005 (0.004)	0.19	drop
	Physical Activity: Mean Vector Magnitude	-0.3 (0.12)	0.02	retain	-0.011 (0.006)	0.048	retain
	15-Month Dietary Diversity in Past 7 Days	0.14 (0.12)	0.27	drop	-	-	-
Maternal BMI							
	Child 6-month Hb	0.10 (0.03)	0.00	retain	0.009 (0.003)	0.01	drop[2]
	Diarrhea Incidence	-0.02 (0.03)	0.46	drop	-	-	-
	Physical Activity: Mean Vector Magnitude	-0.01 (0.03)	0.86	drop	-	-	-
	15-Month Dietary Diversity in Past 7 Days	0.05 (0.03)	0.07	drop	-	-	-
15-Month Dietary Diversity in Past 7 Days							
	Child 6-month Hb	0.06 (0.03)	0.049	retain	0.007 (0.004)	0.07	drop
	Diarrhea Incidence	0.08 (0.03)	0.00	retain	-0.008 (0.004)	0.04	retain
	Physical Activity: Mean Vector Magnitude	-0.04 (0.03)	0.20	drop	-	-	-

[1]Drop due to significant interaction between maternal height and maternal education

[2]Drop due to significant interaction between maternal education and child 6-month Hb

Supplemental Table 10. Pathway Selection Results for Cohort D: ZINC-Burkina Faso

Independent Variable	Potential Mediator	Association between IV and Potential Mediator			Indirect effect in Multiple Mediation Model		
		Estimate (SE)	p-value	Decision	Estimate (SE)	p-value	Decision
Maternal Height (cm)							
	Mother no education	-0.03 (0.04)	0.38	drop	-	-	-
	Unimproved water source	0 (0.04)	0.94	drop	-	-	-
	Maternal BMI	0.02 (0.02)	0.22	drop	-	-	-
	Child 9-month Hb	0.02 (0.02)	0.44	drop	-	-	-
	Change in Hb 9 to 18 months	-0.01 (0.02)	0.45	drop	-	-	-
	Diarrhea incidence	0.04 (0.02)	0.07	drop	-	-	-
	9-month dietary diversity in past 7 days	0.06 (0.02)	0.00	retain	0.005 (0.002)	0.01	retain
	18-Month variety of play materials	0.05 (0.03)	0.09	drop	-	-	-
Mother no education							
	Maternal BMI	0 (0.04)	0.94	drop	-	-	-
	Child 9-month Hb	-0.06 (0.04)	0.14	drop	-	-	-
	Change in Hb 9 to 18 months	-0.03 (0.04)	0.52	drop	-	-	-
	Diarrhea incidence	-0.12 (0.05)	0.01	retain	0.003 (0.003)	0.41	drop
	9-month dietary diversity in past 7 days	-0.07 (0.04)	0.10	drop	-	-	-
	18-Month variety of play materials	-0.32 (0.06)	<.0001	retain	-0.018 (0.008)	0.02	retain
Unimproved water source							
	Maternal BMI	-0.09 (0.04)	0.03	retain	-0.004 (0.004)	0.26	drop
	Child 9-month Hb	-0.1 (0.04)	0.02	retain	-0.003 (0.003)	0.36	drop
	Change in Hb 9 to 18 months	-0.01 (0.04)	0.74	drop	-	-	-
	Diarrhea incidence	0.04 (0.05)	0.44	drop	-	-	-
	9-month dietary diversity in past 7 days	-0.02 (0.04)	0.59	drop	-	-	-
	18-Month variety of play materials	-0.15 (0.07)	0.02	retain	-0.009 (0.005)	0.08	drop
Maternal BMI							
	Child 9-month Hb	0.06 (0.02)	0.00	retain	0.008 (0.006)	0.20	drop
	Change in Hb 9 to 18 months	-0.04 (0.02)	0.048	retain	-0.005 (0.005)	0.29	drop
	Diarrhea incidence	0.05 (0.02)	0.02	retain	-0.008 (0.005)	0.11	drop
	9-month dietary diversity in past 7 days	0.03 (0.02)	0.13	drop	-	-	-
	18-Month variety of play materials	0.07 (0.03)	0.01	retain	0.015 (0.007)	0.03	retain
9-month dietary diversity in past 7 days							
	Child 9-month Hb	0.08 (0.02)	<.0001	retain	0.01 (0.003)	0.00	retain
	Change in Hb 9 to 18 months	-0.02 (0.02)	0.35	drop	-	-	-
	Diarrhea incidence	0.06 (0.02)	0.01	retain	-0.004 (0.002)	0.04997	retain
Child 9-month Hb							
	18-Month variety of play materials	0.14 (0.03)	<.0001	retain	0.028 (0.009)	0.00128	retain
Change in Hb 9 to 18 months							

	18-Month variety of play materials	-0.04 (0.03)	0.24	drop			
Diarrhea incidence							
	18-Month variety of play materials	0.11 (0.04)	0.00	retain	0.017 (0.007)	0.01	retain

Supplemental Table 11. Coefficients for Direct and Indirect Effects on 18-month LAZ in the Final Models

Dependent Variable/ Mediator	Independent Variable	Cohort A: DYAD-Ghana		Cohort B: DYAD-Malawi		Cohort C: DOSE-Malawi		Cohort D: ZINC-Burkina Faso	
		Estimate[a] (SE)	% of effect direct (top) or indirect (bottom)[b]	Estimate[a] (SE)	% of effect direct (top) or indirect (bottom)[b]	Estimate[a] (SE)	% of effect direct (top) or indirect (bottom)[b]	Estimate[a] (SE)	% of effect direct (top) or indirect (bottom)[b]
To LAZ from									
	Maternal Height	0.19*** (0.03)	66%	0.27*** (0.04)	70%	0.28*** (0.03)	98%	0.30*** (0.02)	99%
	Household Asset Index	0.04 (0.03)	72%	-	-	-	-	-	-
	Distance to Nearest Market	-	-	-	-	-0.06* (0.03)	84%	-	-
	Maternal Education	-	-	-	-	0.08** (0.03)	93%	0.06 (0.04)	57%
	Paternal Education	-	-	0.06 (0.04)	100%	-	-	-	-
	Improved Water Source	-	-	-	-	0.11 (0.11)	74%	0.10* (0.04)	100%
	Maternal BMI	-	-	0.03 (0.03)	38%	0.10*** (0.03)	94%	0.10*** (0.02)	92%
	Maternal Hb at < 20 weeks gestation	0.07* (0.03)	100%	-	-	-	-	-	-
	9- or 15-Month dietary diversity in past 7 days	-	-	-	-	0.02 (0.02)	128%	0.06** (0.02)	92%
	18-Month variety of play materials	-	-	0.03 (0.03)	100%	-	-	0.14*** (0.03)	100%
	18-Month activities with caregivers	-	-	0.10* (0.03)	100%	-	-	-	-
	Gestational age at birth	0.17*** (0.03)	100%	0.13** (0.04)	94%	-	-	-	-
	LGAZ at birth	0.51*** (0.03)	100%	0.45*** (0.04)	100%	-	-	-	-
	Child 6 or 9-month Hb Concentration	-	-	0.05 (0.05)	100%	0.06* (0.03)	100%	0.12*** (0.03)	88%
	Change in Hb 6 or 9 to 18 months	-	-	0.04 (0.05)	100%	-	-	0.13*** (0.03)	100%
	Child 18-month AGP	-	-	-0.15*** (0.04)	100%	-	-	-	-
	Diarrhea incidence 6 or 9 to 18 months	-	-	-	-	-0.09** (0.03)	100%	-0.10*** (0.02)	113%

Prevalence of reported poor appetite (% of days)	-0.04 (0.03)	100%	-	-	-	-	-	-
Accelerometer Mean Vector Magnitude	-	-	-	-	0.13*** (0.03)	100%	-	-
To Maternal BMI from								
Maternal Education	-	-	-	-	0.06* (0.03)	7%	-	-
Distance to Nearest Market	-	-	-	-	-0.13*** (0.03)	11%	-	-
To Maternal Hb at < 20 weeks gestation from								
Household Asset Index	0.20*** (0.03)	17%	-	-	-	-	-	-
To 9-month dietary diversity in past 7 days from								
Maternal Height	-	-	-	-	-	-	0.06** (0.02)	1%
To 18-Month variety of play materials from								
Maternal Education	-	-	-	-	-	-	-0.31*** (0.06)	43%
Maternal BMI	-	-	-	-	-	-	0.06* (0.03)	8%
Child 9-month Hb	-	-	-	-	-	-	0.12*** (0.03)	12%
Diarrhea incidence 9 to 18 mo	-	-	-	-	-	-	0.08* (0.04)	-8%
To Gestational age at birth from								
Household Asset Index	0.10** (0.03)	11%	-	-	-	-	-	-
To LGAZ at birth from								
Maternal Height	0.23*** (0.03)	34%	0.26*** (0.04)	30%	-	-	-	-
Maternal BMI	-	-	0.11* (0.04)	62%	-	-	-	-
To Child 6 or 9-month Hb Concentration from								
Maternal Height	-	-	-	-	0.08* (0.03)	2%	-	-
Maternal BMI	-	-	-	-	0.10*** (0.03)	6%	-	-
9-Month dietary diversity in past 7 days	-	-	-	-	-	-	0.08*** (0.02)	12%
Gestational age at birth	-	-	0.19*** (0.04)	6%	-	-	-	-
To Diarrhea incidence 6 or 9 to 18 months from								

Distance to Nearest Market	-	-	-	-	0.10*** (0.03)	5%	-	-
9- or 15-Month dietary diversity in past 7 days	-	-	-	-	0.06** (0.02)	-28%	0.06** (0.02)	-5%
To Accelerometer Mean Vector Magnitude from								
Improved Water Source	-	-	-	-	0.29* (0.12)	26%	-	-

[a]Estimates are standardized such that they represent the change in units of SD of the dependent variable/mediator for each 1 SD change in the independent variable.

[b]In the top half of the table, under the heading "Direct Effects" the percentage represents the proportion of the effect of the independent variable that is directly associated with the dependent variable. In the bottom half of the table, under the heading "Indirect Effects" the percentage represents the proportion of the effect of the independent variable on the dependent variable that is mediated by the mediator.

*$p < 0.05$ after correcting for multiple comparisons using the Benjamini Hochberg correction
**$p < 0.01$ after correcting for multiple comparisons using the Benjamini Hochberg correction
***$p < 0.001$ after correcting for multiple comparisons using the Benjamini Hochberg correction

References

1. Adu-Afarwuah S, Lartey A, Okronipa H, et al. Small-quantity, lipid-based nutrient supplements provided to women during pregnancy and 6 mo postpartum and to their infants from 6 mo of age increase the mean attained length of 18-mo-old children in semi-urban Ghana: a randomized controlled trial. *American journal of clinical nutrition* 2016
2. Adu-Afarwuah S, Lartey A, Okronipa H, et al. Small-quantity, lipid-based nutrient supplements provided to women during pregnancy and 6 mo postpartum and to their infants from 6 mo of age increase the mean attained length of 18-mo-old children in semi-urban Ghana: a randomized controlled trial. *Am J Clin Nutr* 2016;104(3):797-808. doi: 10.3945/ajcn.116.134692
3. Ashorn P, Alho L, Ashorn U, et al. Supplementation of Maternal Diets during Pregnancy and for 6 Months Postpartum and Infant Diets Thereafter with Small-Quantity Lipid-Based Nutrient Supplements Does Not Promote Child Growth by 18 Months of Age in Rural Malawi: A Randomized Controlled Trial. *J Nutr* 2015;145(6):1345-53. doi: 10.3945/jn.114.207225
4. Hess SY, Abbeddou S, Jimenez EY, et al. Small-quantity lipid-based nutrient supplements, regardless of their zinc content, increase growth and reduce the prevalence of stunting and wasting in young Burkinabe children: a cluster-randomized trial. *PLoS One* 2015;10(3):e0122242. doi: 10.1371/journal.pone.0122242
5. Maleta KM, Phuka J, Alho L, et al. Provision of 10-40 g/d Lipid-Based Nutrient Supplements from 6 to 18 Months of Age Does Not Prevent Linear Growth Faltering in Malawi. *J Nutr* 2015;145(8):1909-15. doi: 10.3945/jn.114.208181
6. Vyas S, Kumaranayake L. Constructing socio-economic status indices: how to use principal components analysis. *Health Policy and Planning* 2006;21(6):459-68. doi: 10.1093/heapol/czl029
7. Coates J, Swindale A, Bilinsky P. Household Food Insecurity Access Scale (HFIAS) for Measurement of Household Food Access: Indicator Guide (v. 3). Washington, D.C: Food and Nutrition Technical Assistance Project, Academy for Educational Development 2007.
8. Cohen S, Kamarck T, Mermelstein R. A global measure of perceived stress. *J Health Soc Behav* 1983;24(4):385-96.
9. Stewart CP, Oaks BM, Laugero KD, et al. Maternal cortisol and stress are associated with birth outcomes, but are not affected by lipid-based nutrient supplements during pregnancy: an analysis of data from a randomized controlled trial in rural Malawi. *BMC Pregnancy Childbirth* 2015;15(1):346. doi: 10.1186/s12884-015-0793-8
10. Cox JL, Holden JM, Sagovsky R. Detection of postnatal depression: Development of the 10-item Edinburgh Postnatal Depression Scale. *British Journal of Psychiatry* 1987;150:782-86.
11. WHO. A user's guide to the Self-Reporting Questionnaire (SRQ). Geneva: World Health Organization 1994.
12. Stewart RC, Kauye F, Umar E, et al. Validation of a Chichewa version of the self-reporting questionnaire (SRQ) as a brief screening measure for maternal depressive disorder in Malawi, Africa. *Journal of Affective Disorders* 2009;112(1-3):126-34. doi: 10.1016/j.jad.2008.04.001 [published Online First: 2008/05/28]
13. Villar J, Ismail LC, Victora CG, et al. International standards for newborn weight, length, and head circumference by gestational age and sex: the Newborn Cross-Sectional Study of the INTERGROWTH-21st Project. *The Lancet* 2014;384(9946):857-68. doi: 10.1016/S0140-6736(14)60932-6
14. Pulakka A, Cheung YB, Ashorn U, et al. Feasibility and validity of the ActiGraph GT3X accelerometer in measuring physical activity of Malawian toddlers. *Acta Paediatr* 2013;102(12):1192-8. doi: 10.1111/apa.12412 [published Online First: 2013/10/10]

15. Trost SG, Fees BS, Haar SJ, et al. Identification and validity of accelerometer cut-points for toddlers. *Obesity (Silver Spring)* 2012;20(11):2317-9. doi: 10.1038/oby.2011.364
16. World Health Organization. Indicators for assessing infant and young child feeding practices. Part 2 Measurement. Geneva: WHO 2010.
17. Kariger P, Frongillo EA, Engle P, et al. Indicators of family care for development for use in multicountry surveys. *J Health Popul Nutr* 2012;30(4):472-86.
18. Hamadani JD, Tofail F, Hilaly A, et al. Use of family care indicators and their relationship with child development in Bangladesh. *J Health Popul Nutr* 2010;28(1):23-33.
